# Alterations in 3D chromatin organization contribute to tumorigenesis of *EGFR*-amplified glioblastoma

**DOI:** 10.1016/j.csbj.2022.04.007

**Published:** 2022-04-08

**Authors:** Qi Yang, Nian Jiang, Han Zou, Xuning Fan, Tao Liu, Xi Huang, Siyi Wanggou, Xuejun Li

**Affiliations:** a Department of Neurosurgery, Xiangya Hospital, Central South University, No. 87, Xiangya Road, Changsha, Hunan 410008, PR China; b Hunan International Scientific and Technological Cooperation Base of Brain Tumor Research, Xiangya Hospital, Central South University, No. 87, Xiangya Road, Changsha, Hunan 410008, PR China; c Annoroad Gene Tech. (Beijing) Co., Ltd, Block 1, Yard 88, Kechuang 6 RD, Beijing Economic-Technological Development Area, Beijing 100176, PR China; d Program in Developmental and Stem Cell Biology, The Hospital for Sick Children, Toronto, Ontario, M5G 1X8, Canada; e Arthur and Sonia Labatt Brain Tumour Research Centre, The Hospital for Sick Children, Toronto, Ontario, M5G 1X8, Canada; f Department of Molecular Genetics, University of Toronto, Toronto, Ontario, M5S 3E1, Canada

**Keywords:** Hi-C, Glioblastoma, Astrocytes, Structure Variation, Tumorigenesis, 3D genomic, WGS, Whole-genome sequencing, TAD, Topological associating domain, SV, Structure variation, GBM, Glioblastoma multiform, PCC, Pearson’s correlation coefficient, ICE, Iterative correction and eigenvector decomposition, IDE, Distance decay exponent, DE, Differentially expressed, FDR, False discovery rate

## Abstract

**Background:**

*EGFR* amplification and/or mutation are found in more than half of the cases with glioblastoma. Yet, the role of chromatin interactions and its regulation of gene expression in *EGFR*-amplified glioblastoma remains unclear.

**Methods:**

In this study, we explored alterations in 3D chromatin organization of *EGFR-*amplified glioblastoma and its subsequent impact by performing a comparative analysis of Hi-C, RNA-seq, and whole-genome sequencing (WGS) on *EGFR*-amplified glioblastoma-derived A172 and normal astrocytes (HA1800 cell line).

**Results:**

A172 cells showed an elevated chromatin relaxation, and unexpected entanglement of chromosome regions. A genome-wide landscape of switched compartments and differentially expressed genes between HA1800 and A172 cell lines demonstrated that compartment activation reshaped chromatin accessibility and activated tumorigenesis-related genes. Topological associating domain (TAD) analysis revealed that altered TAD domains in A172 also contribute to oncogene activation and tumor repressor deactivation. Interestingly, glioblastoma-derived A172 cells showed a different chromatin loop contact propensity. Genes in tumorigenesis-associated signaling pathways were significantly enriched at the anchor loci of altered chromatin loops. Oncogene activation and tumor repressor deactivation were associated with chromatin loop alteration. Structure variations (SVs) had a dramatic impact on the chromatin conformation of *EGFR-*amplified glioblastoma-derived tumor cells. Moreover, our results revealed that 7p11.2 duplication activated *EGFR* expression in *EGFR*-amplified glioblastoma via neo-TAD formation and novel enhancer-promoter interaction emergence between *LINC01446* and *EGFR*.

**Conclusions:**

The disordered 3D genomic map and multi-omics data of *EGFR-*amplified glioblastoma provide a resource for future interrogation of the relationship between chromatin interactions and transcriptome in tumorigenesis.

## Highlights

• There is widespread chromatin disorganization in EGFR-amplified glioblastoma.
• Chromatin disorganization contribute to tumorigenesis in glioblastoma.
• Structural variations have a substantial impact on chromatin conformation.

## Graphical abstract

**Figure:**
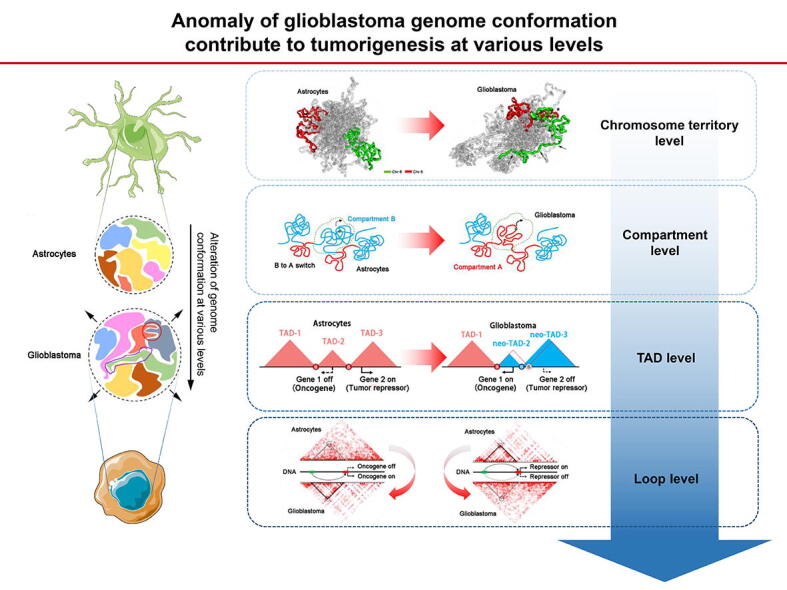

## 1 Introduction

Glioblastoma multiform (GBM) is a highly malignant brain tumor. Its median overall survival time is approximately one year upon diagnosis [1], [2]. Molecular subtype not only reflects inter-tumor heterogeneity, but also have impact on the overall survival of glioblastoma patients. *EGFR* amplification and/or mutation occurs in more than half of cases. *EGFR*-related signaling pathways are over-stimulated [3], [4], [5], resulting in malignant characteristics of cancer cells and poor prognosis for these patients [6]. Unfortunately, the results of clinical trials of *EGFR*-targeted therapies have not been encouraging [7], [8]. Therefore, it is necessary to explore tumorigenesis mechanisms and discover novel therapeutic targets for this type of brain tumor.

Cancer cells accumulate various genomic structure and epigenetic alterations during tumorigenesis. The 3D genomic organization is disordered, and its alterations at levels of the compartment, topologically associating domains (TADs), or chromatin loop that regulates long-range enhancer-promoter interactions and activates oncogene or deactivates tumor repressor [9], [10]. Previously, research on *IDH* mutant glioma revealed that disrupted TAD boundary activates key oncogene expression programs [11]. The aberrant long-range interactions between enhancer elements and their target genes point toward the possibility of identifying new glioma therapy [12], [13]. However, the role of chromatin interactions and its regulation of gene expression in *EGFR*-amplified glioblastoma remains unclear.

To elucidate 3D genome structure alteration and its possible consequences in *EGFR*-amplified glioblastoma, in this study, we performed a comparative analysis of Hi-C, RNA-seq, and whole-genome sequencing (WGS) on *EGFR*-amplified glioblastoma-derived A172 and normal astrocytes (HA1800 cell line). This disordered 3D genomic map and multi-omics data of malignant *EGFR*-amplified glioblastoma provide a resource for future interrogation of the relationship between epigenetic and genetic in tumorigenesis.

## 2 Materials and Methods

### 2.1 Cell culture

A172 cells were obtained from ATCC, maintained in DMEM with 10% FBS and 1% Plasmocin (InvivoGen) at 37 °C in a 5% CO2 environment. HA1800 cells obtained from ScienCell were grown in Astrocyte Medium (ScienCell, Cat. #1801) with 10% FBS at 37 °C in a 5% CO2 environment. HA1800 cells were harvested for *in situ* Hi-C at 90% confluence at the third or fourth passage. A172 cells were harvested for *in situ* Hi-C at 90% confluence.

### 2.2 *In situ* Hi-C library preparation

Method for *in situ* Hi-C library preparation in this study derived from Rao, S. S. et. al. [14] with minor modifications. About 5 × 10^6^ cells (per 100 mm plate) were harvested with 22.5 mL serum free fresh medium and crosslinked by formaldehyde (1.25 mL of 37% formaldehyde) at 2% final concentration in the plate for 10 min at room temperature (RT). After which, 2.5 mL of 2.5 M glycine was added to the mixture in order to quench the crosslinking reaction, incubate for 5 min at RT and then incubate on ice for 15 min. Scrape the cells from the plates and transfer to a tube. 5 plates of cells were pooled to prepare 1 library before sequencing. Centrifuge the crosslinked cells at 800 xg for 10 min and discard the supernatant. Wash the pallet with ice-cold 1x PBS and centrifuge at 300 xg at 4℃ for 5 min, discard the supernatant and flash-freeze the pallet in liquid nitrogen.

Wash the pellet by resuspending it in 500 μL of ice-cold Hi-C lysis buffer (10 mM Tris-HCl pH8.0, 10 mM NaCl, 0.2% Igepal CA630, 1x protease inhibitors cocktail), incubate on ice for 20 min and then centrifuging the sample for 5 min at 2500 xg. Then wash the pallet with Hi-C lysis buffer again. The nuclei were washed by 0.5 mL of CutSmart buffer (NEB #B7204S) and transferred to a safe-lock tube. Next, the chromatin is solubilized with dilute SDS and incubation at 65℃ for 10 min. After quenching the SDS by Triton X-100. Overnight digestion was applied with 4-cutter restriction enzyme (400 units MboI) at 37℃ on rocking platform.

The next steps are Hi-C specific, including marking the DNA ends with biotin-14-dCTP and performing blunt-end ligation of crosslinked fragments. The proximal chromatin DNA was religated by ligation enzyme. The nuclear complexes were reversed crosslinked by incubating with proteinase K at 65℃. DNA was purified by phenol–chloroform extraction. Biotin-C was removed from non-ligated fragment ends using T4 DNA polymerase. Fragments was sheared to a size of 200–600 base pairs by sonication. The fragment ends were repaired by the mixture of T4 DNA polymerase, T4 polynucleotide kinase and Klenow DNA polymerase. Biotin labeled Hi-C sample were specifically enriched using streptavidin C1 magnetic beads. The fragment ends were adding A-tailing by Klenow(exo-) and then adding Illumina paired-end sequencing adapter by ligation mix. At last, the Hi-C libraries were amplified by 12–14 cycles PCR, and sequenced in Illumina HiSeq-2500. Sequencing interacting patterns were obtained by Illumina HiSeq-2500 instrument with 2 × 150-bp reads.

### 2.3 Preprocess of Hi‐C datasets

Raw reads of Hi-C data was processed by HiC-Pro (v2.11.1) pipeline [15] using the bowtie2 end-to-end algorithm with default parameters. Unmapped paired-end reads, singleton reads, multiple mapped reads and PCR duplication were filtered, only uniquely valid paired-end reads were kept for downstream analysis (Table S1). All 12 libraries were separately processed and quality checked, we observed high correlation among libraries (Fig. S1A, B) which indicating the high quality and reproducibility of the dataset, therefore, valid paired-end reads of each 6 libraries of A172 were then merged into one to improve resolution of the matrices, same by HA1800. To determine the highest resolution of our dataset, we used the method by Rao, S. S. et. al. [14]. As shown in Figure S1E, the 20th quantile of per bin contact count binned at 5 kb for both cell lines are over 1000, indicates the input dataset can at least reach 5 kb in resolution. Subsequent raw contact matrices are produced at all resolutions (5 kb, 10 kb, 40 kb, 50 kb, 100 kb, 200 kb, 200 kb, 500 kb, 1 Mb) for further analysis. ICE (iterative correction and eigenvector decomposition) [16], a robust bias removal technique built into HiC-Pro was used for normalization of raw contact matrices. Further analysis was based on normalized matrix unless stated. Default parameters are used for all analyses unless otherwise specified.

### 2.4 RNA-seq, WGS library preparation

A total of 6 RNA-seq libraries (3replicationsforeachcellline) were prepared using NEBNext® Ultra™ RNA Library Prep Kit for Illumina® (#E7530L, NEB, USA) by the instructions of the manufacture. A total of 2 WGS libraries (1 for each cell line) were prepared with TruSeq DNA Sample Prep Kit by the instructions of the manufacture. All libraries were sequenced in Illumina HiSeq-2500 with paired-ends 2 × 150-bp reads. Sequencing statistics indicate acceptable quality (Table S2, S3).

### 2.5 RNA-seq, WGS data analysis

RNA-seq data was mapped to human reference genome (hg19) by HISAT2 (v2.1.0) [17] with default parameters, aligned reads were then quantified by featureCounts software [18], differential expression analysis was performed by DESeq2 [19].

WGS data was mapped to human reference genome (hg19) by BWA-MEM [20] with default parameters, aligned reads were then processed by GATK4 pipelines for somatic SNPs [21], structural variations (duplications, inversions, deletions, translocations) were called by delly (v0.7.5) [22], copy number variations were called by Control-FREEC [23].

### 2.6 Identification of A/B compartment profiles and translocation events

With matrix2compartment.pl script in cworld-dekker software (v1.01) available through GitHub (https://github.com/dekkerlab/cworld-dekker), intra‐chromosomal Hi‐C matrices at a resolution of 500 kb was used to identify Compartment A/B. TAD boundary was identified using intra‐chromosomal Hi‐C matrices at 50 kb resolution using matrix2insulation.pl in cworld-dekker software. As matrices for the each of the two cell lines were pooled from six separate libraries, we checked the correlation of the 1st eigenvector value (Fig. S2A, B) and insulation score (Fig. S2C, D) between all 12 libraries. Each of the 6 libraries shown moderately high intra-group consistency, no matter for A172 and HA1800. IDEs were performed using intra‐chromosomal Hi‐C heatmaps at 500 kb with matrix2scaling.pl in cworld-dekker software. Translocation events were identified using inter-chromosomal Hi-C matrices at 40 kb with HiCtrans [24] (v2.0). We then find all double-confirmed translocation event by the following criteria, the edge of Hi-C identified translocation contains breakpoint called from WGS. All double-confirmed translocation events (Table S7) were masked from further analysis unless stated otherwise.

### 2.7 Identification of chromatin loops

Loops are identified using Juicer [25] (v1.6.2) with default parameters on intra‐chromosomal Hi‐C matrices at 10 kb. Specific loop is defined as the loops that are unique by both anchors for a cell line.

### 2.8 3D genome modeling

3D reconstruction of chromosome conformation was performed with inter- (at 1 Mb resolution) and intra-chromosomal (at 50 kb resolution) Hi-C matrices, using Chrom3D [26] (v1.0.1) by the instructions in the manual using default parameters with minor modifications. For A172, in order to alleviate the effects of inter-chromosome translocation, any inter-chromosome interaction overlapping with translocated regions confirmed by both WGS and HiCtrans were purged before modeling. Significant inter-chromosome interactions were call with FDR = 0.1 in HA1800 to retain restrains of chromosome 15, instead of default value of 0.01 which was used in A172. For 3D genome modelling of 6 single libraries of each cell (Fig. S4B, C), FDR threshold for significant intra- and inter-chromosome interaction was set to 0.2.

## 3 Results

### 3.1 The altered chromatin structure of *EGFR-*amplified glioblastoma shows increased distance from nuclear periphery to the center, elevated chromatin relaxation, and unexpected entanglement of chromosome territories.

**Fig. 1:**
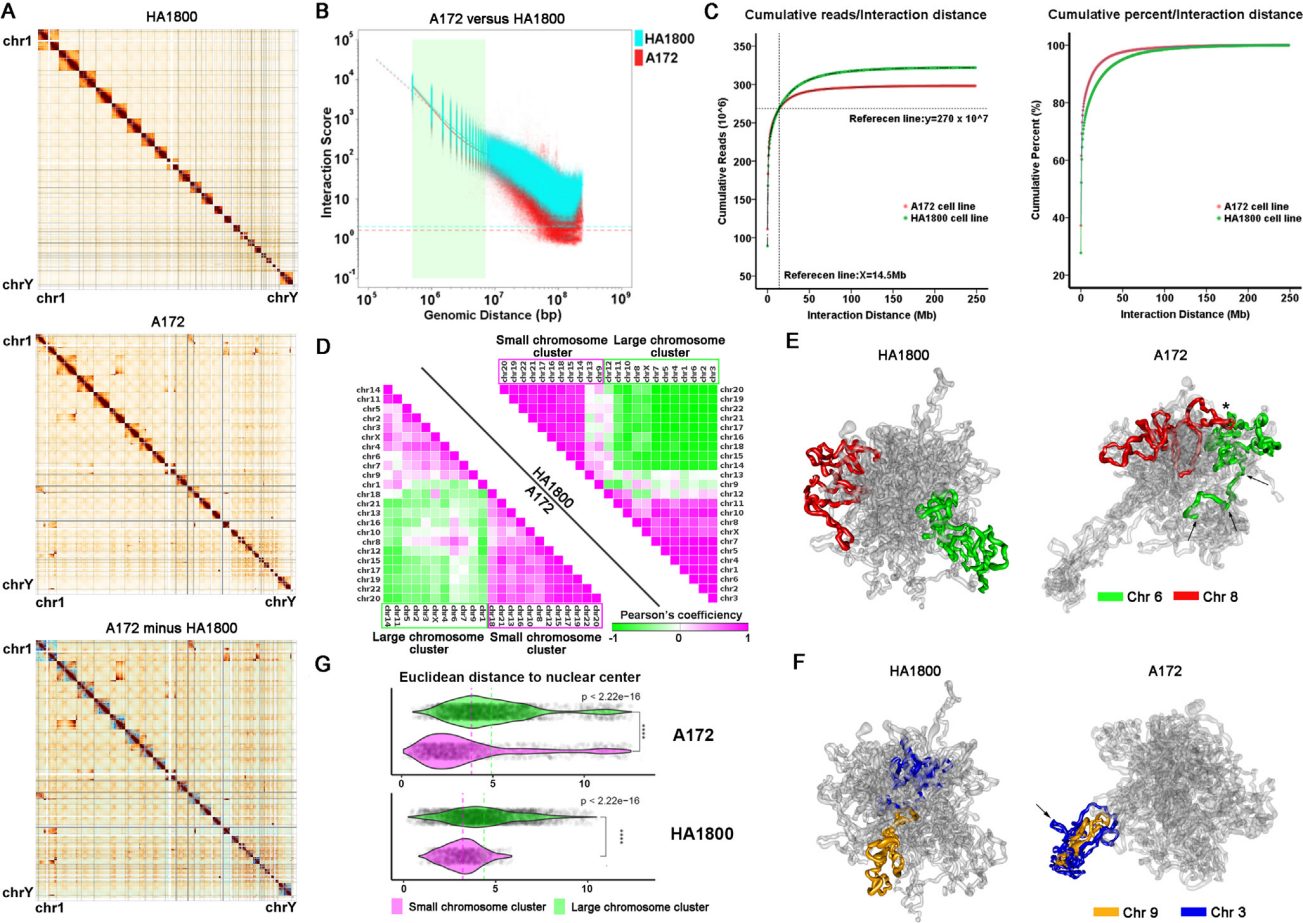
(A) ICE normalized contact maps at 500-kb resolution of HA1800 (top), A172 (middle) and A172 minus HA1800 (bottom). (B) Merged intra-chromosome IDE curve of A172 and HA1800 on all chromosomes. (C) Cumulative intra-chromosome interactions by genomic distance of A172 and HA1800 (left). Cumulative percentage of intra-chromosome interaction by genomic distance of A172 and HA1800 (right). (D) Pearson correlation coefficient matrix of collapsed inter-chromosome contact map from HA1800 and A172. Chromosomes are grouped by unsupervised hierarchical clustering. (E) 3D reconstruction of relative spatial distribution of chromatin based on Hi-C data. Chromatin relaxation of chromosome 6 and chromosome 8 in A172 (arrows). Asterisk shows chromosome territories entanglement of chromosome 6 and chromosome 8. (F) 3D reconstruction of relative spatial distribution of chromatin based on Hi-C data. Chromosome territories entanglement of chromosome 9 and chromosome 13 in A172. (G) Comparison of Euclidean distance to nuclear center between small and large chromosome clusters in HA1800 (left) and A172 (right). Dots represent individual TADs. Dotted lines represent mean value of each group. Non-parametric Wilcoxon test was used for statistics.

We generated contact maps up to 5 kb resolution by pooling 6 Hi-C libraries of each cell line together (Fig. 1A). Unbiased clustering showed high similarity within the two cell lines of the 12 libraries with different amount of interaction pairs and sequencing depth that passed our initial quality control process (Fig. S1B, C). Pearson’s correlation coefficients (PCCs) of all libraries exceed 0.7 within the cell line, and inter-cell line PCCs were slightly lower at 0.6 (Fig. S1A, B). All libraries had dominantly more *cis*-interactions than *trans*-interactions, which is consistent with the current understanding of chromosome territories formation (Fig. S1C, top). The moderate similarity between normal astrocytes and *EGFR*-amplified glioblastomas was deeply embedded in the chromatin structure, and the differences between them might reveal the reason for tumorigenesis.

Iterative correction and eigenvector decomposition (ICE) normalized contact map (Fig. 1A, top) of normal astrocyte HA1800 cell line (referred to as HA1800 in the following context) shows a typical plaid pattern of a mammalian cell characterized with much greater intra-chromosome interactions than inter-chromosome interactions [27]. Compared with HA1800, the contact map of the *EGFR*-amplified glioblastoma-derived A172 cell line (referred to as A172 in the following context) showed a much “noisier” background (Fig. 1A, middle & bottom). The ratio of *trans*-interactions significantly increased in A172 (Fig. S1C, bottom, Table S1). Interestingly, A172 showed overall increased inter-chromosome interactions around centromeres, resulting from centromere clusters formation during active mitosis [28].

It is well accepted that intra-chromosome interactions decay by power-law when genomic distance gradually increases between two loci in a Hi-C contact map of human cells [29], [30]. In general, *cis*-interactions decayed faster in A172 at the whole genome scale, indicating a more isolated genomic structure (Fig. 1B). Despite differences in sequencing depth, *cis*-interactions cumulate faster in A172 by distance, not only in absolute number but also in percentage (Fig. 1C). In general, the distance decay exponents (IDEs) profiles were similar between A172 and HA1800 in chromosomes 15–17 and 19–22 (Fig. S3C). This might be a result of intrinsic active and stable transcription on these gene-rich and centripetal chromosomes. Interestingly, we observed curved-up tails on the IDEs graph in HA1800 at both whole genome-scale and individual chromosome-scale (Fig. S3B, C), while such tails were either lower or even reversed in A172 (Fig. S3A, C). The IDE findings suggested that telomeres at both sides of the same chromosome in A172 were physically less proximal than HA1800. Per-chromosome IDEs could be roughly split into three segments, 0.1–1 Mb, 1–10 Mb, and 10- Mb, based on the characteristics of the graph (Fig. S3C). In the range of < 1 Mb that corresponds to TADs [31], [32], A172 has a greater interaction frequency over HA1800, the gap is then smoothed in the range of 1–10 Mb, and finally reversed in the range of > 10 Mb where A172 has less intra-chromosome interactions. Intra-chromosome interactions spanning 10 Mb are unlikely to be transcription-activating in function and more likely to be the organizer of the higher-order globular chromatin architecture. The loss of intra-chromosome interactions and the increased inter-chromosome interactions of A172 add the possibility of physical contact between chromosomes, which might cause increased genome instability and translocation events in the cancer genome.

Next, we performed hierarchical clustering of collapsed inter-chromosome interaction maps at a resolution of 500 kb (Fig. S1D). The Pearson’s co-efficiency heatmap showed that two major chromosome clusters were mutually exclusive with each other, thus suggesting the spatial gathering tendency of large and small chromosomes was irrelevant (Fig. 1D). In HA1800, the heatmap showed a clear and obvious boundary between large and small chromosome clusters, suggesting highly ordered chromatin organization of normal astrocytes. Interestingly, in A172, such order was disrupted by chromosome 6 and chromosome 8 (Fig. 1D). To visualize the difference, we performed a 3D reconstruction based on a Hi-C contact map (Fig. S4A). As a result, the A172 genome showed an irregular spatial organization, compared with HA1800. Obviously, we observed clear chromatin relaxation of chromosome 6 and chromosome 8 in A172 (Fig. 1E, arrow, Fig. S4B, C), resulting in elevated collapsed inter-chromosome interaction on these two chromosomes (Fig. 1D). Unexpectedly, we observed increased physical inter-chromosome entanglement (Fig. 1E. asterisk, Fig. S4B, C). Also, such neo-chromosome entanglement could be observed between chromosome 3 and chromosome 9 in A172 (Fig. 1F, arrow, Fig. S4B, C). In general, genome structure is much more relaxed and entangled in A172, as demonstrated in the 3D reconstruction (Fig. S4 A) and contact map (Fig. 1A) of the Hi-C data.

During interphase, compartmentalized chromosomes can be clustered into two groups that corresponding to nuclear location, nuclei, and periphery. Inter-group chromosome translocation can bring some active chromosome arms to inactive zone and vice versa. It has been reported [33] that gene-rich chromosomes, such as chromosomes 15–17 and 19–22, tend to be located in the center of the nucleus. In A172 and HA1800, two groups of chromosomes showed different locations (Fig. 1G). Large chromosomes were further away from small chromosomes in A172 compared to HA1800 (Fig. 1G), suggesting a trend of increasing polarization.

### 3.2 B to A compartment switch contributes to oncogene activation in *EGFR-*amplified glioblastoma

**Fig. 2:**
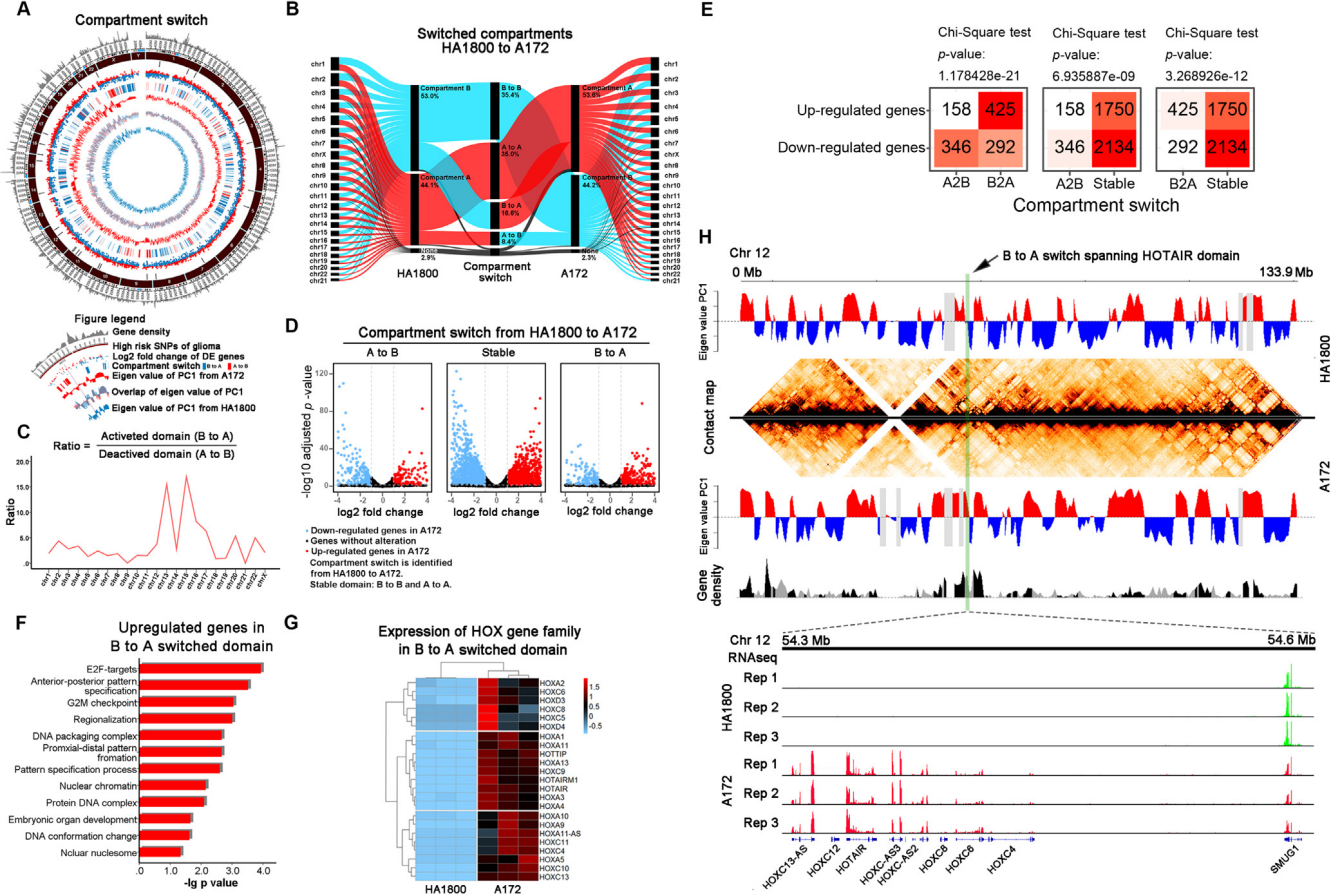
(A) Genome-wide landscape of switched compartments and differentially expressed genes from HA1800 to A172. (B) Proportion of compartments and compartment switch patterns from HA1800 to A172. (C) Ratio between activated domains (B to A switch) and deactivated domains (A to B switch) in each chromosome. (D) Differentially expressed genes in activated (B to A switch), deactivated (A to B switch) and stable domains (A to A or B to B) between HA1800 and A172. (E) Contingency tables for compartment switch and differential gene expression between the two cell lines. A2B, A-to-B compartment switch: A in HA1800 switched to B in A172. B2A, B-to-A compartment switch: B in HA1800 switched to A in A172. Up-regulation means the gene has significantly higher expression in A172, and vice versa. (F) Enriched GO pathways of upregulated genes at B-to-A switched domains. (G) Differentially expressed *HOX* gene family at B-to-A switched domains. (H) Contact map and compartment comparison on chromosome 12 between HA1800 and A172. Arrow shows B-to-A compartment switch spanning *HOTAIR* domain. Magnified RNA-seq shows gene expression in this domain.

Compartment A and B are generally polarized in spatial position within a single chromosome or whole genome-wide. Compartment A is normally oriented towards nuclei and genes and has a higher transcription activity, while compartment B stays near the nuclear periphery and is more adherent to the nuclear envelope [14], [29], [34], [35]. Compared to HA1800, about 16.6% of the whole genome “flipped” from B compartment to A compartment in A172, and only 8.4% “flipped” from A to B (Fig. 2A, B). From HA1800 to A172, the “activated” compartment switches (B to A) were more pervasive than “deactivated” manner (A to B), resulting in dramatic changes in gene expression (Fig. 2B). Such a compartment switch manner could be observed in almost all the single chromosomes (Fig. S5A-D). We defined an activation ratio as the length of domains that are “activated” divided by the “deactivated” (Fig. 2C). At compartment level, top activated chromosomes like 13, 15, 16, 17, 20, and 22 in A172 belongs to the centripetal group as indicated by previous chromosome location analysis (Fig. 1D). The altered relative spatial position might be the cause of abnormal B-to-A compartment switch in A172. Notably, chromosome 13 locates in a peripheral group of HA1800 but becomes the one with the largest proportion of A compartment and locates in a centripetal group of A172.

To investigate the impacts of compartment switch at the transcriptomic level, we analyzed gene expression of HA1800 and A172 under three switching states (Fig. 2D, E). In stable compartment, the ratio between up-regulated and down-regulated gene is 1750/2134 = 0.820. In A-to-B switched compartment, this ratio is 158/346 = 0.457, indicating significantly more genes than expected are down-regulated from HA1800 to A172, by Chi-Square test. While in B-to-A switched compartment, this ratio is 425/292 = 1.455, indicating significantly more genes than expected are up-regulated from HA1800 to A172 (Fig. 2E). The results above showed that compartment switch can exert a contributory but not deterministic role on transcription regulation. To investigate the concept that B to A compartments switch activates oncogene expression, we performed enrichment analysis focusing on up-regulated genes in B-to-A switched domains. The enrichment analysis revealed that these genes were majorly enriched in anterior-posterior pattern specification, regionalization, proximal–distal pattern formation, embryonic organ development, DNA conformation change, and cell cycle control, all of which are considered to accelerate tumor growth (Fig. 2F).

Interestingly, we noticed that in B-to-A switched domains, *HOX* gene family was generally activated (Fig. 2G). As a result, *HOX* gene family was taken as an example to illustrate the relationship between compartment switch and oncogene activation. Members of *HOX* gene family were oncogenic in glioma and many other solid tumors. Up-regulated *HOX* genes are essential to glioblastoma growth by regulating multiple pathways and are usually related to poor survival. Over-expression of *HOTAIRM1* up-regulates *HOXA1,* which then increases the invasiveness of glioblastoma [36]. *HOXA5* increases cell proliferation and radiation resistance [37]. *HOXC10* up-regulates *VEGFA* by binding to its promoter, thus promoting angiogenesis [38]. *HOXC10* also activates immunosuppressing genes like *PD-L2* and *TDO2* by direct binding to their promoters [39]. In this study, we selected *HOX* genes that locate in these “activated” B-to-A switched domains for further analysis. Concordantly, their expression was significantly elevated in A172 (Fig. 2G). *HOX* gene family was clustered on chromosomes. We then took the cluster spanning *HOTAIR* as an example. This region on chromosome 12 (start from 54.3 Mb to 54.6 Mb) was in B-to-A switched domain and was “reactivated” in A172 (Fig. 2H). RNA-seq analysis confirmed that in the same region, *HOX* genes were activated (Fig. 2G). “Activation” of gene-sparse regions like *HOX* clusters demonstrated that compartment switch could serve as an important regulator for oncogene transcription program initiation.

### 3.3 TAD boundary alteration is associated with comprehensive activation of oncogenes and deactivation of tumor suppressors in *EGFR-*amplified glioblastoma

TAD boundaries are generally accepted as tissue-specific genome insulators in mammals that separate transcription activities in neighboring domains [27]. TAD boundary alterations have been shown to be oncogenic by affecting gene expression [9]. There are two basic types of TAD boundary alterations: disappearance and emergence. TAD boundary disappearance can cause unwanted gene upregulation due to exposure to originally isolated enhancers in neighboring TADs, while TAD boundary emergence could turn down gene expression by isolating functional enhancers.

**Fig. 3:**
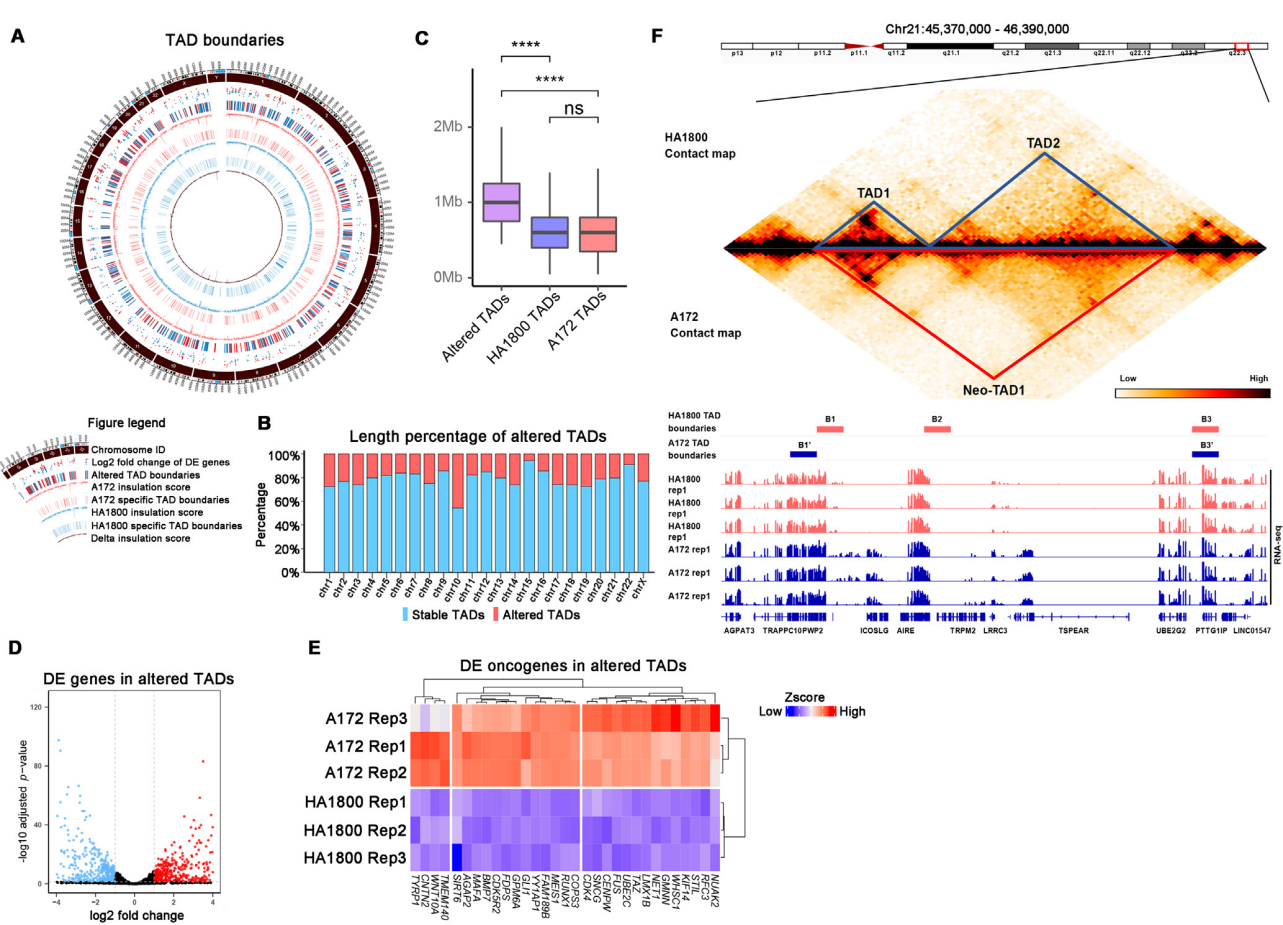
(A) Genome-wide landscape of insulation score, TAD boundaries and differentially expressed genes in HA1800 and A172. Insulation score and TAD boundaries were inferred with contact map at 50 kb. (B) Proportion of stable and altered TADs between HA1800 and A172 in each chromosome. (C) Boxplots for TAD size in A172, HA1800 and altered TADs between them. Wilcoxon test was used for statistics. **** refer to *p* value < 0.0001. (D) Volcano plot of Differentially expressed genes between HA1800 and A172 in altered TADs. (E) Differentially expressed oncogenes in altered TADs between HA1800 and A172 in altered TADs. (F) Contact map, TAD boundaries and RNA-seq in altered TAD domain spanning *TRPM2*.

To investigate the TAD alteration pattern of *EGFR-*amplified glioblastoma, we compared the TAD boundaries of A172 and HA1800 (Fig. 3A). Interestingly, almost the same number of TAD boundaries was found in the two cells (4075 and 4085, respectively). By comparing TAD boundary domains, we generated altered boundaries and TADs, HA1800 specific versus A172 specific. A172 has 380 specific TAD boundaries, while HA1800 has 421 specific ones (Fig. S6A). We then calculated the percentage of DNA length in altered TADs versus full DNA length in each chromosome and found that the TADs are generally in stable status (Fig. 3B), suggesting minor 3D genomic alteration is responsible for tumorigenesis in glioblastoma. Consequently, we compared the TAD size and found that the size distribution of all TADs was stable in both A172 and HA1800 (Fig. S6B), with 600 kb as the median. Interestingly, TADs with altered boundaries were significantly larger in size (Fig. 3C). Most chromosomes were relatively stable from HA1800 to A172 (Fig. S6A), with roughly 20% of its length covered with unstable TADs (Fig. 3B). Chromosome 10 was conspicuously the most unstable one, with nearly half of its total length covered by unstable TADs, while chromosome 15 was the most stable one (Fig. 3B). The insulation score represents how many inter-TAD interactions cross a given bin (genome divided into fixed-length); a lower score means stronger insulation. Therefore, TADs boundaries are identified as valleys on the curve of insulation score along a chromosome. A similar appearance on insulation score curves of chromosome 10 between A172 and HA1800 reflected conservation of chromatin structure at TAD level (Fig. S6A). We then subtracted the insulation score of A172 by HA1800. Fluctuations on the curve indicate the complexity of TAD boundary alterations. Except for the easily identifiable position shifting of boundaries, the insulation strength of TAD boundaries also changed from A172 to HA1800 (Fig. 3A, Fig. S6A). Unfortunately, little is known about the impact of altered insulation strength on the function of TAD boundaries. To elucidate the effect of TAD boundary alteration, we investigated one SVs-free region on chromosome 21, from 45.37 Mb to 46.37 Mb (Fig. 3F). The boundary B2 of HA1800 vanished in A172, resulting the merging of TAD1 and TAD2 into neo-TAD1, consequently increasing inter-TAD interaction and expression of *TRPM2, ICOSLG and TSPEAR* (Fig. 3F). Previous reports indicated that *TRPM2*, *ICOSLG* are potential oncogenes in glioma [40], [41].

To further explore the global impact of TAD boundary alterations on tumorigenesis, we analyzed all known oncogenes and tumor suppressors located in unstable TADs. Surprisingly, 31 oncogenes in altered TADs showed significantly higher expression in A172 (Fig. 3D, E). Among these oncogenes, overexpression of *NUAK2* promotes proliferation and invasion of A172 [42],*GLI1* is a well-known glioma-associated transcription factor; suppression of *TMEM140* attenuates growth of glioma cell [43]. On the other hand, 36 tumor suppressors were down-regulated in altered TAD of A172 (Fig. S6C). For most of these genes, the relationship with glioblastoma has not been elucidated and needs further exploration.

### 3.4 Chromatin looping depicts a bi-directional regulation of oncogenes and tumor suppressors in *EGFR-*amplified glioblastoma

Chromatin looping is the basic chromatin structure that represents the spatial proximity of two genomic loci and enables enhancers to interact with distal targets. A complicated looping structure can also form “hub” like structure to simultaneously facilitate transcription of multiple genes.

**Fig. 4:**
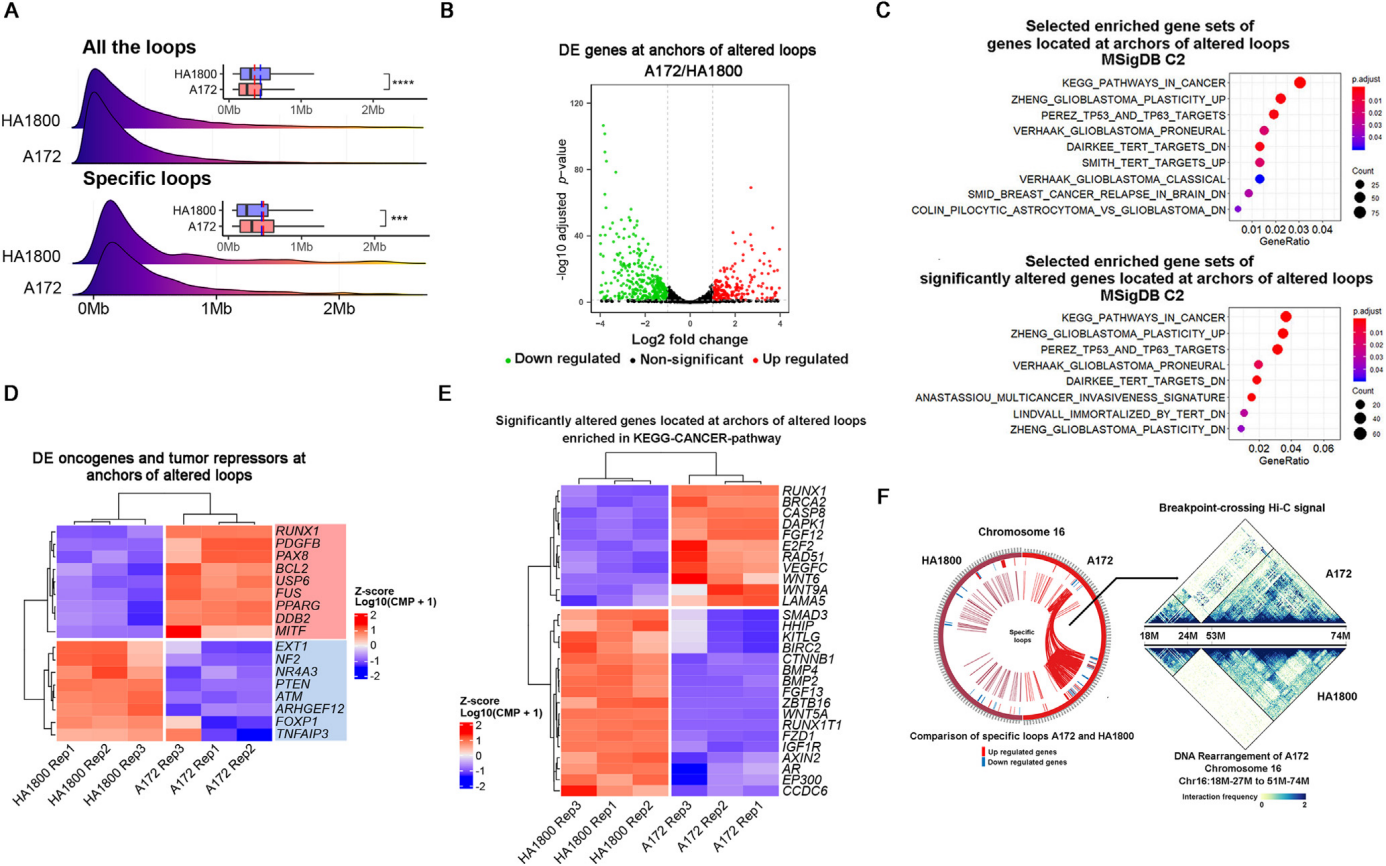
(A) Length comparison of all the loops in HA1800 and A172. Ridge plots show loop length distribution. Bar plots show statistics. Solid lines show median length. Dotted lines show average length. Wilcoxon test was used for statistics. **** refer to a *p* value < 0.0001. *** refer to a *p* value < 0.001. (B) Differentially expressed genes located at anchors of altered loops. (C) Enriched pathways of genes at anchors of altered loops (Upper). Enriched pathways of differentially expressed genes at anchors of altered loops (Lower). (D) Differentially expressed oncogenes (in the red rectangle) and tumor repressors (in the blue rectangle) at anchors of altered loops. (E) Transcriptome profiles of differentially expressed genes located at anchors of specific loops enriched in KEGG-CANCER-pathway. (F) Specific loops on chromosome 16 of A172 and HA1800, contact map of the region from 18 Mb to 27 Mb and the region from 51 Mb to 74 Mb.

To explore the alteration of 3D genomic structure at loop level in *EGFR*-amplified glioblastoma, we analyzed the Hi-C data from both HA1800 and A172 cell lines. From chromosome 1 to X, chromatin loops are evenly distributed (Fig. S7D), and the loop count of each chromosome is proportional to its length in both cell lines (Fig. S7A). HA1800 has significantly more loops than A172 for all chromosomes except for chromosome 16 (Fig. S7A). Interestingly, the length span of loops in HA1800 is also significantly longer (Fig. 4A), corresponding well with the fact described above that TADs with altered boundaries are larger in size. The numbers of specific loops (altered loops between the two cell lines) that only occur in either A172 or HA1800 on each chromosome are almost identical, despite the huge difference in chromosome length with one exception (Fig. S7B). Chromosome 16 has the most and highest ratio of specific loops in A172, and it is also the only chromosome on which A172 has more loops than HA1800 (Fig. S7B, C). Nearly half of the loops on chromosome 16 are specific to A172. We noticed that chromosome 16 has intra-chromosome rearrangement events (Fig. 4F); the region from 18 Mb to 27 Mb swapped with the region from 51 Mb to 74 Mb. Such intra-chromosome rearrangement events transformed the chromatin conformation of chromosome 16 in A172.

Further enrichment analysis of genes located at anchors of specific loops (Fig. 4B) illustrates how chromatin loops at the transcription level define cancer cells. Among the nine pathways that have the FDR (false discovery rate) of < 0.05 (Fig. 4C, upper), four are directly related to glioblastoma, the rest include general cancer pathways, *TERT*-related pathways, all of which are determinate factors of glioblastoma malignant behavior. Enrichment analysis results were similar when we further analyzed differentially expressed genes located at anchors of specific loops (Fig. 4C, lower). These genes were involved in pathways directly related to glioblastoma, invasiveness signature, TERT-related, and general cancer pathways. Transcription profiles of the genes enriched in the KEGG-CANCER-pathway showed distinct states between A172 and HA1800 (Fig. 4E). Differentially expressed oncogenes and tumor repressors at the anchors of altered chromatin loops had distinct expression patterns in both cell lines (Fig. 4D), thus suggesting chromatin loop alterations have a dual role in tumorigenesis.

### 3.5 Genome structure variations remodel chromatin conformation and contribute to the enhanced expression of *EGFR*

**Fig. 5:**
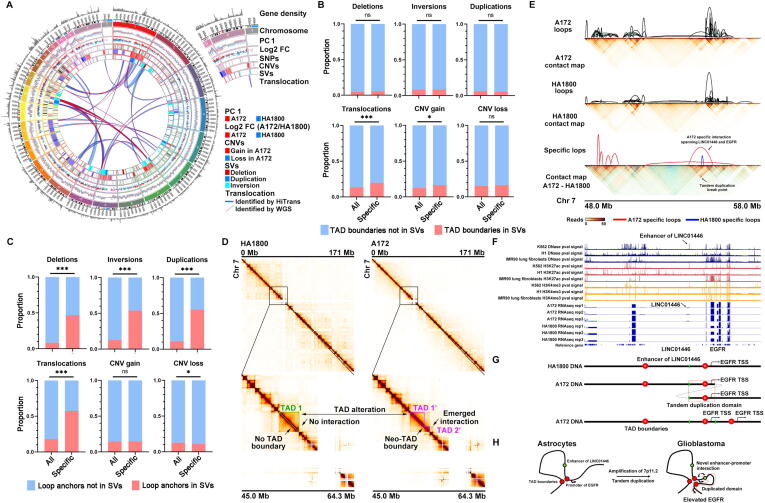
(A) Genomic landscape of chromosomes in A172 cell line. Compartment associated PC1 eigenvalue, log2 fold change of differentially expressed genes, loci of SNPs, DNA domains of copy number variations (CNVs) and structure variations (SVs), and DNA translocation events are integrated. (B) The proportion of TAD boundaries affected by structure variations between all boundaries and altered boundaries. The Chi-square test was used for statistics. *** represents a *p*-value < 0.001, * represents *a p*-value < 0.05, ns represents *a p*-value > 0.05. (C) The proportion of loop anchors affected by structure variations between all anchors and altered anchors. The Chi-square test was used for statistics. *** represents a *p*-value < 0.001, * represents *a p*-value < 0.05, ns represents a *p*-value > 0.05. (D) Hi-C contact map of chromosome 7 in HA1800 (left) and A172 (right). DNA domains from 45.0 Mb to 64.3 Mb are magnified. Contact maps show obvious neo-TAD boundary and emerged interactions spanning chromosome 7p11.2. TAD in HA1800 is framed by green, and neo-TADs in A172 are labeled by fuchsia. The resolution of the contact map is 50 kb. (E) Magnified contact map and loop comparison between A172 and HA1800, centering EGFR from chr7 48.0 Mb to 58.0 Mb. The arrow shows A172 specific interaction connecting LINC01446 and EGFR. A Tandem duplication breakpoint is identified by whole-genome sequencing (WGS). (F) DNase signal, H3K27ac signal, H3K4me3 signal of K562, H1, and IMR90 cell lines help to identify enhancer of LINC01446. RNA-seq of HA1800 and A172 reveals altered expression of LINC01446 and EGFR. (G) Schematic of the locus in HA1800 and A172 (top and middle). LINC01446 enhancer is labeled in green; the Tandem duplication domain is labeled in the red frame. Linear DNA schematic (bottom) shows neo-TAD in A172. TSS, transcription start site. (H) Working model of 7p11.2 duplication activates EGFR expression in EGFR amplified glioblastoma by novel enhancer-promoter interaction connecting LINC01446 and EGFR via neo-TAD.

Chromosome translocation can be identified from Hi-C data [24]. Tools like HiCtrans can be used to explore inter-chromosome translocation events by detecting abnormal *trans*-interaction hotspots in the whole contact map. Nevertheless, the accuracy of the translocation boundaries is pre-defined by the resolution of the contact map. In this study, we identified translocation events more accurately with the aid of WGS because WGS with adequate sequencing depth can pinpoint the exact breakpoints on the whole genome. Translocation events reported by HiCtrans were manually checked, and valid translocations were included in the final results when at least one border was confirmed by breakpoints identified with WGS (Fig. 5A, Table S5). The preferences of chromosomes that these translocation events locate indicate inter-chromosomal proximity [44], high frequent translocation events of A172 between chromosomes 1, 2, 7, 9, 16, and X might be a result of chromothripsis and deviated chromosome compartmentalization.

Somatic genome structure variations could cause dramatic changes to cancer cells in the perspective of chromatin conformation. To evaluate its possible effect, we used WGS data of both cell lines to identify somatic SVs in A172. Then, we compared the counts of loops and TAD boundaries that are located in or out of SVs in A172. Our data showed that SVs include deletion, inversion, duplications, translocations, and CNV loss, thus affecting the chromatin conformation at the looping level (Fig. 5C, Table S6). About half of specific loops in A172 have at least one anchor in regions with deletion, inversion, duplications, or translocations. Contrary, only a small amount of all loops have anchors within such regions. Unexpectedly, CNV gain did not seem to affect loops, and there were significantly lesser specific loops in the region with CNV loss. The effect of somatic SVs was weaker at the level of TAD boundaries. Moreover, a significantly higher proportion of specific TAD boundaries of A172 were observed only in SVs, including translocation and CNV gain (Fig. 5B, Table S6). SVs disrupted the sequential arrangement of genomic loci, and increased the chance of interaction that should not exist. This might be the reason why loops are more affected by SVs, while TAD boundary is thought to be partially predefined by the DNA sequence. Therefore, inside the migrated DNA fragment, TAD boundary relocates in the genome without apparent damage of its insulating power.

Next, we examined how the chromatin conformation was affected by the amplification of 7p11.2 on chromosome 7 and whether such chromatin conformation contributes to enhanced *EGFR* expression. Interestingly, by comparing A172 with HA1800, we observed obvious TAD and chromatin interaction alterations at the region of 7p11.2 (Fig. 5D). There was a clear emergence of neo-TAD boundary splitting the normal TAD1 in HA1800 into two separated TADs in A172 (Fig. 5D). By subtracting the contact map spanning 7p11.2, an additional chromatin interaction occurred in A172 and was identified as tandem duplication (Fig. 5E). This tandem repeats together with the emerging TAD boundary, greatly reshaped the chromatin landscape around *EGFR*. By subtracting the chromatin loops, we identified an A172 specific interaction connecting *LINC01446* and *EGFR* (Fig. 5E). By combining ENCODE DNase sequencing data and H3K27ac/H3K4me3 ChIPseq data, we identified the possible epigenetic activation signals of *LINC01446* and *EGFR* (Fig. 5F)*.* Additional RNA-seq analysis confirmed the elevated expression of *LINC01446* and *EGFR* in A172, suggesting the co-activation of both two gene loci (Fig. 5F). Together with WGS, we reconstructed the linear model of emerged enhancer-promoter interaction spanning *LINC01446* and *EGFR* by 7p11.2 tandem duplications (Fig. 5G). The duplicated 7p11.2 domain includes *LINC01446* on one side and *EGFR* on the other. When this duplicated domain repeats after the original one, physical proximity of *LINC01446* and *EGFR* occurs. These A172 specific long-distance TAD-spanning loops weave the region into a chromatin hub with an additional enhancer-promoter interaction connecting *LINC01446* and *EGFR*, which enables the enhancer of nearby *LINC01446* to upregulate *EGFR* expression (Fig. 5H).

### 3.6 Altered chromatin conformation at various levels have substantial impact on transcription regulation in glioblastoma

**Fig. 6:**
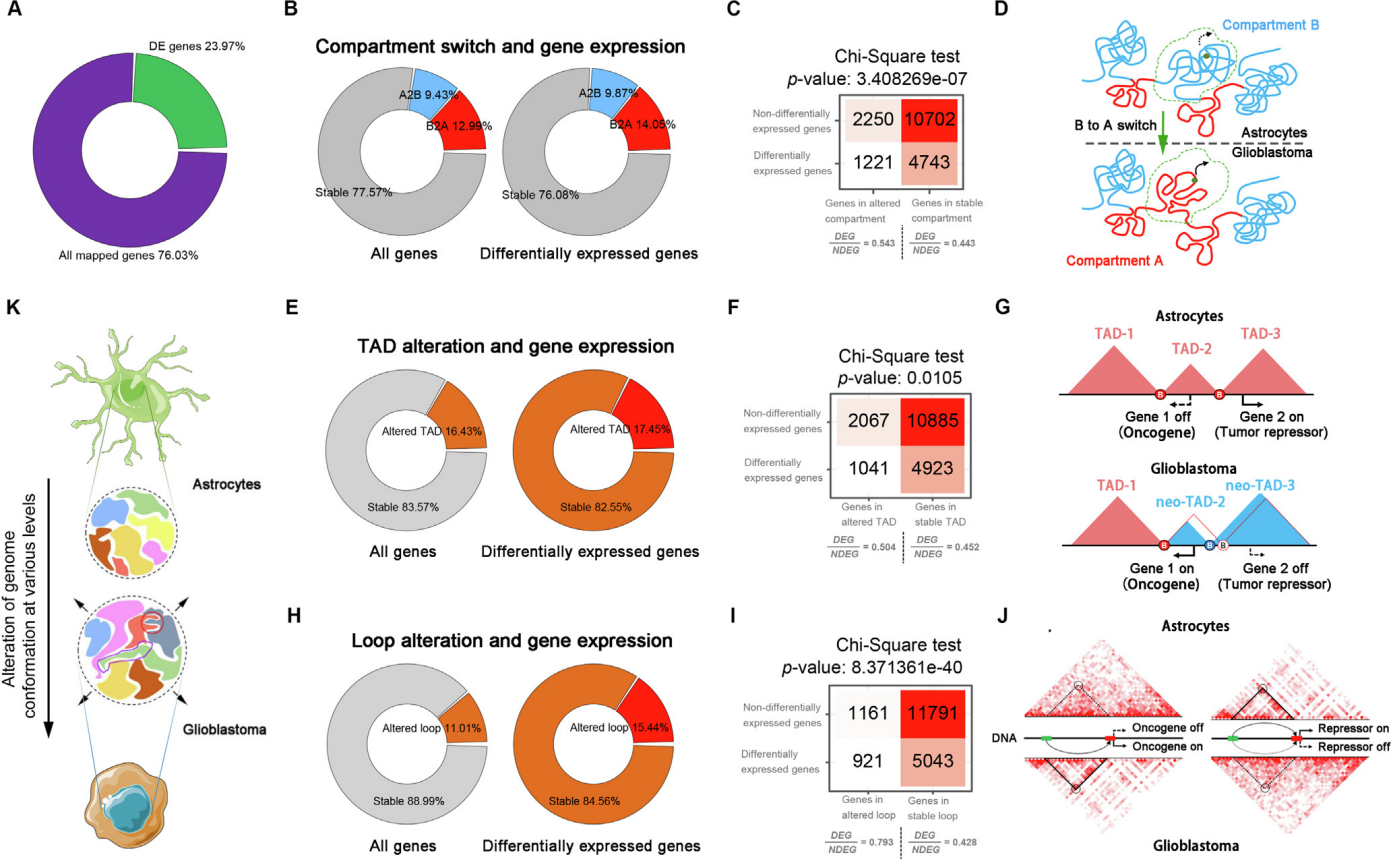
(A) The proportion of differentially expressed genes among all mapped genes in RNA-seq. (B) The proportion of genes at activated, deactivated and stable compartment domains (left). The proportion of differentially expressed genes at different compartment domains (right). A2B, compartment A-to-B switch from HA1800 to A172. B2A, compartment B-to-A switch from HA1800 to A172. (C) Contingency tables for compartment switch and differential gene expression between the two cell lines. DEG, differentially expressed gene. NDEG, non-differentially expressed gene. (D) Schematic of compartment activation in glioblastoma. (E) Gene distribution in stable and altered TADs (left). The proportion of differentially expressed genes in stable and altered TADs (right). (F) Contingency tables for TAD alteration and differential gene expression between the two cell lines. DEG, differentially expressed gene. NDEG, non-differentially expressed gene. (G) Neo-TADs, oncogene activation and tumor repressor deactivation in glioblastoma. Alteration of TAD boundaries (disappearing, emerging or shifting) emerges neo-TADs in glioblastoma, contributing to oncogene activation and tumor repressor deactivation. (H) The proportion of genes located at anchors of altered loops in whole-genome (left). The proportion of differentially expressed genes in genes at anchors of altered loops (right). (I) Contingency tables for loop alteration and differential gene expression between the two cell lines. DEG, differentially expressed gene. NDEG, non-differentially expressed gene. (J) Loop alteration, oncogene activation, and tumor repressor deactivation in glioblastoma. Neo-loop emerging activates oncogene expression (left), and the disappeared loop deactivates tumor repressor (right). (K) Schematic of major findings in this study. Compared with chromatin conformation of astrocytes, glioblastoma shows alterations at various levels.

From HA1800 to A172, 23.97% of all mapped genes are differentially expressed (Fig. 6A). 77.57% of all mapped genes locate in “stable” domains in terms of compartment switch. And the majority of differentially expressed (DE) genes were also located in regions without compartment switch (Fig. 6B). To investigate the effect of compartment switch on transcriptional regulation, we compared the ratio between differentially expressed genes (DEGs) and non-differentially expressed genes (NDEGs) (Fig. 6C). The elevated DEG/NDEG ratio from stable to altered compartment indicates that compartment switch can provide some explanations to the altered transcriptomic profile in A172 (Fig. 6D). For TAD boundary alteration, there are 83.57% of all mapped genes and 82.55 % of DEGs locate in stable TADs (Fig. 6E). Similar to the results of compartment switch, an elevated DEG/NDEG ratio is also observed in altered TADs (Fig. 6F). Among all significantly differentially expressed genes between A172 and HA1800, only 17.45% were located at anchors of altered loops (Fig. 6H). But loop alteration resulted in most dramatic DEG/NDEG ratio change, compared with compartment switch or TAD alterations (Fig. 6I). These findings indicate that chromatin loop contributes most to transcriptional regulation.

Based on the results above, we propose a chromatin confrontation associated transcriptional regulation model at three different genomic levels: compartments, TADs and loops. In tumorigenesis, B-to-A compartment switch reshapes the chromatin accessibility in glioblastoma and is associated with activation of tumorigenesis-related genes (Fig. 6D). TAD boundary shifting, disappearance or emergence, reshape the neighboring domains, resulting in the formation of neo-TADs associated with oncogene activation and tumor suppressor suppression (Fig. 6G). In addition, we propose a bi-directional regulation model of EGFR-amplified glioblastoma: the emergence of neo-loop around oncogenes contributed to the activation of the oncogenic transcription program in A172 (Fig. 6J, left) while disappearing or weakening of existing loop around tumor suppressor crippled the anti-tumor line of defense in HA1800 (Fig. 6J, right).

## 4 Discussion

In this study, we discovered widespread chromatin organization alterations of *EGFR-*amplified glioblastoma at the compartment, TAD, and loop levels, which contribute to oncogene activation and tumor repressor deactivation in *EGFR-*amplified glioblastoma. We also identified glioma-specific long-range interactions. Genes located in these regions tend to be more activated. Moreover, enrichment analysis revealed that genes regulated by gliomas-specific long-range interactions are enriched in *PI3K*-*AKT*, Ras signaling pathways, and *EGFR* tyrosine kinase inhibitor resistance, all of which are associated with malignancy of GBM. Genomic structure variation drastically impacts chromatin conformation in A172 cells. Finally, we found out that tandem duplication of the *EGFR* domain results in the formation of neo-TAD and novel enhancer-promoter interaction between *LINC01446* and *EGFR,* which contributes to the elevation of *EGFR* expression.

Analysis at the scale of tens of Mb, compartments of chromosomes, illustrated the instability of the tumor genome, especially on gene-rich small chromosomes. About a quarter of the total genome went through compartment switch. Up-regulated genes located in “activated” domains are enriched in pathways associated with mitosis and pattern specification process. Compartment switch from B to A is associated with over-expression of *HOX* genes in A172. *HOX* family can promote the growth of glioblastoma [36], [37], [45], [46]). With regard to sub-Mb structures, TADs are overall stable both in numbers and sizes between the two cell lines. Nevertheless, alterations of insulating elements lead to increased TADs size in A172 cells. Exposure to *cis*-regulatory elements in adjacent but originally isolated TADs may lead to positive regulation of genes, as demonstrated by expressions of oncogenes in unstable TADs. On the other hand, the emergence of neo-boundary can reduce the expression of tumor suppressors [9]. We also observed widespread, varied insulation strength of TAD boundaries between A172 and HA1800; however, their effect still remains unclear. Spatial proximity between enhancer and promoter is necessary for transcription initiation in human cells [47], [48], [49]. Chromatin loop per se can induce gene expression, but it is not sufficient [50], [51]. Organized chromatin conformation might be a result of transcription regulation other than the cause [52], [53]. In this work, we found out that a relatively high proportion of DE genes locate at anchors of specific loops, indicating chromatin topology is moderately associated with tumorigenesis of glioblastoma.

Hi-C and WGS could work in synergy to improve accuracy in the detection of translocation events. Somatic SVs like translocations have dramatic influences on the cancer genome in the perspective of chromatin conformation [54]. Transcription factor binding sites carried within SVs can create chances for the faulty formation of loops. TAD boundaries are less affected by SVs, which could be explained by relatively larger domains that are intrinsically more stable [35]. Amplification of 7p11.2 is an important characteristic of *EGFR*-amplified glioblastoma [4], [5], but how this amplification affects chromatin conformation in glioblastoma remains unknown. We identified an A172 specific loop that reshaped this amplified region into a chromatin hub. The reshaped chromatin conformation in glioblastoma cells contributed to the upregulation of *EGFR* by exploiting enhancers from flanking TAD.

In HA1800 and A172 cells, 23.92% of DE genes are located in domains with compartment switch, while 17.45% of them are located in domains with altered TAD boundaries, which is slightly higher than previous reports on terminal differentiation [52]. Moreover, 15.44% of them were located at anchors of specific loops. On the contrary, chromatin loop alteration have highest DEG/NDEG ratio, indicating a finer and stronger control of genes in cancer cell, compared with compartment switch and TAD alteration. All these alterations contribute to the tumorigenesis in *EGFR*-amplified glioblastoma. Future studies are needed to further explain the interplay between genetic, epigenetic, 3D genome, and transcription for specific oncogenes in *EGFR*-amplified glioblastoma.

In summary, our analysis above depicts an interesting transition of genome structure between normal astrocytes and *EGRF*-amplified glioblastoma, from compacted to unconsolidated, from compartmented to farraginous. The astrocytes probably suffer such transition during tumorigenesis to the *EGFR*-amplified glioma cells (Fig. 6K). The altered genome conformation at various levels have substantial impact on the transcriptomic profile of A172. In the process of tumorigenesis. Compartment switch and TAD provides regulation within large genomic domain yet have weaker effect, chromatin looping have strong influence on transcriptome but only for a small proportion of genes (Fig. 6D, G, J).

## 5 Conclusions

The 3D chromatin organization of the *EGFR-*amplified glioblastoma-derived A172 genome is altered at various levels. Remodeling of cancer genome during tumorigenesis occurs at all hierarchy of genome folding, contributes to the oncogenic transcription program, and cripples the tumor suppressors. Somatic SVs in the cancer genome have a substantial impact on chromatin conformation, especially on loops. *EGFR* duplication creates neo-TAD and novel enhancer-promoter interaction between *LINC01446* and *EGFR,* which elevates *EGFR* expression. This is the first multi-omics dataset comparing histologically homologous *EGFR*-amplified glioblastoma with astrocytes, which provides a valuable resource for future of the relationship between chromatin interactions and transcriptome in tumorigenesis.

## Ethics approval and consent to participate

This research was approved by the Ethics Committee of the Xiangya Hospital Central South University.

## Availability of data and materials

The processed data and code of current study are available from the corresponding author on reasonable request. The raw data generated and/or analyzed during the current study are available in the Sequence Read Archive (SRA) under the accession number PRJNA532762.

## CRediT authorship contribution statement

**Qi Yang:** Methodology, Investigation, Software, Writing – original draft, Visualization, Data curation. **Nian Jiang:** Methodology, Investigation, Data curation. **Han Zou:** Software, Formal analysis, Visualization. **Xuning Fan:** Formal analysis, Visualization. **Tao Liu:** Visualization, Project administration. **Xi Huang:** Conceptualization, Writing – review & editing, Funding acquisition. **Siyi Wanggou:** Conceptualization, Methodology, Software, Funding acquisition, Software, Writing – original draft, Data curation. **Xuejun Li:** Conceptualization, Methodology, Writing – review & editing, Funding acquisition, Supervision, Project administration, Resources.

## Declaration of Competing Interest

The authors declare that they have no known competing financial interests or personal relationships that could have appeared to influence the work reported in this paper.
