# Supplementary material for: Alterations in 3D chromatin organization contribute to tumorigenesis of EGFR-amplified glioblastoma
Source: Comput Struct Biotechnol J. 2022 Apr 8;20:1967–78. doi: 10.1016/j.csbj.2022.04.007 (PMC9062087; doi:10.1016/j.csbj.2022.04.007)
Supplement: Supplementary data 1 [file mmc1.pdf]

Supplementary Figure

Figure S1. (A) The upper five are smooth scatter plots of Pearson correlation of ICE normalized interaction frequency between the first library and the rest 5 libraries of A172; "R" in the plot stands for Pearson correlation coefficient. The lower five are the same plots for HA1800. **(B)** Heatmap of Pearson correlation coefficient matrix of 12 libraries. **(C)** Statistics of valid interaction pairs generated from 12 libraries. The proportion of cis/trans interaction pairs of all 12 libraries. **(D)** Heatmaps of A172 (Upper two). From left to right: the first one is the collapsed inter-chromosome contact map at 500-kb resolution with hierarchical clustering, the second one is the Jaccard index matrix of the previous contact map. Heatmaps of HA1800 (Lower two). **(E)** Histogram of the contact count per bin of 5kb Hi-C matrices of HA1800 (Upper, blue) and A172 (Lower, red). Vertical black lines denote the 20th quantile of contact count.

Figure S2. (A) Heatmap showing the value of the 1st eigenvector of 500kb Hi-C matrices of all 12 libraries generated in this study. The 1st eigenvector of 500kb Hi-C matrices were used to call A/B compartment. **(B)** Correlation of the 1st eigenvector of 500kb Hi-C matrices of all 12 libraries generated in this study. **(C)** Heatmap showing the insulation score of 50kb Hi-C matrices of all 12 libraries generated in this study. The insulation score of 50kb Hi-C matrices were used to call TAD boundaries in this study. **(D)** Correlation of the insulation score of 50kb Hi-C matrices of all 12 libraries generated in this study.

Figure S3. (A) Intra-chromosome IDE curve of A172 on all chromosomes. **(B)** Intra-chromosome IDE curve of HA1800 on all chromosomes. **(C)** Merged intra-chromosome IDE curve of A172 and HA1800 per chromosome.

Figure S4. (A) 3D reconstruction of the genome of A172 (Both on original data and translocation-masked data) and HA1800 based on Hi-C data. **(B)** 3D reconstruction of the genome of every single library of A172 (Translocation-masked). **(C)** 3D reconstruction of the genome of every single library of HA1800 (Translocation-masked).

Figure S5. (A) The percentage that compartment A and B occupy in HA1800 per chromosome. **(B)** The percentage that compartment A and B occupy in A172 per chromosome. **(C)** The percentage of two types of A/B compartment switch (A to B, B to A) accounting for HA1800 to A172 per chromosome. "activated": switched from B to A; "deactivated": switched from A to B; "stable": no compartment switch; "none": undetermined compartment. **(D)** Compartment switch profile of all chromosomes.

Figure S6. (A) Insulation score profile and TAD boundaries of all chromosomes. Legends of stacked plot of each chromosome (from top to bottom): A172 contact map, A172 insulation score, A172 TAD boundaries, HA1800 contact map, HA1800 insulation score, HA1800 TAD boundaries, contact map of A172 minus HA1800, insulation score of A172 minus HA1800, altered TAD boundaries from A172 to HA1800. For TAD boundaries and curve of insulation score; red indicates A172 while green indicates HA1800. **(B)** Density distribution of TAD size in A172, HA1800 and altered TADs between them. **(C)** Differentially expressed tumor suppressors in altered TADs.

Figure S7. (A) Histogram of numbers of loops identified per chromosome in A172 and HA1800. **(B)** Histogram of numbers of specific loops identified per chromosome in A172 and HA1800. **(C)** Histogram of radio that specific loops account for all loops in A172 and HA1800. **(D)** Atlas of chromatin loops for all chromosomes of A172 and HA1800.

Sup. Figure 1

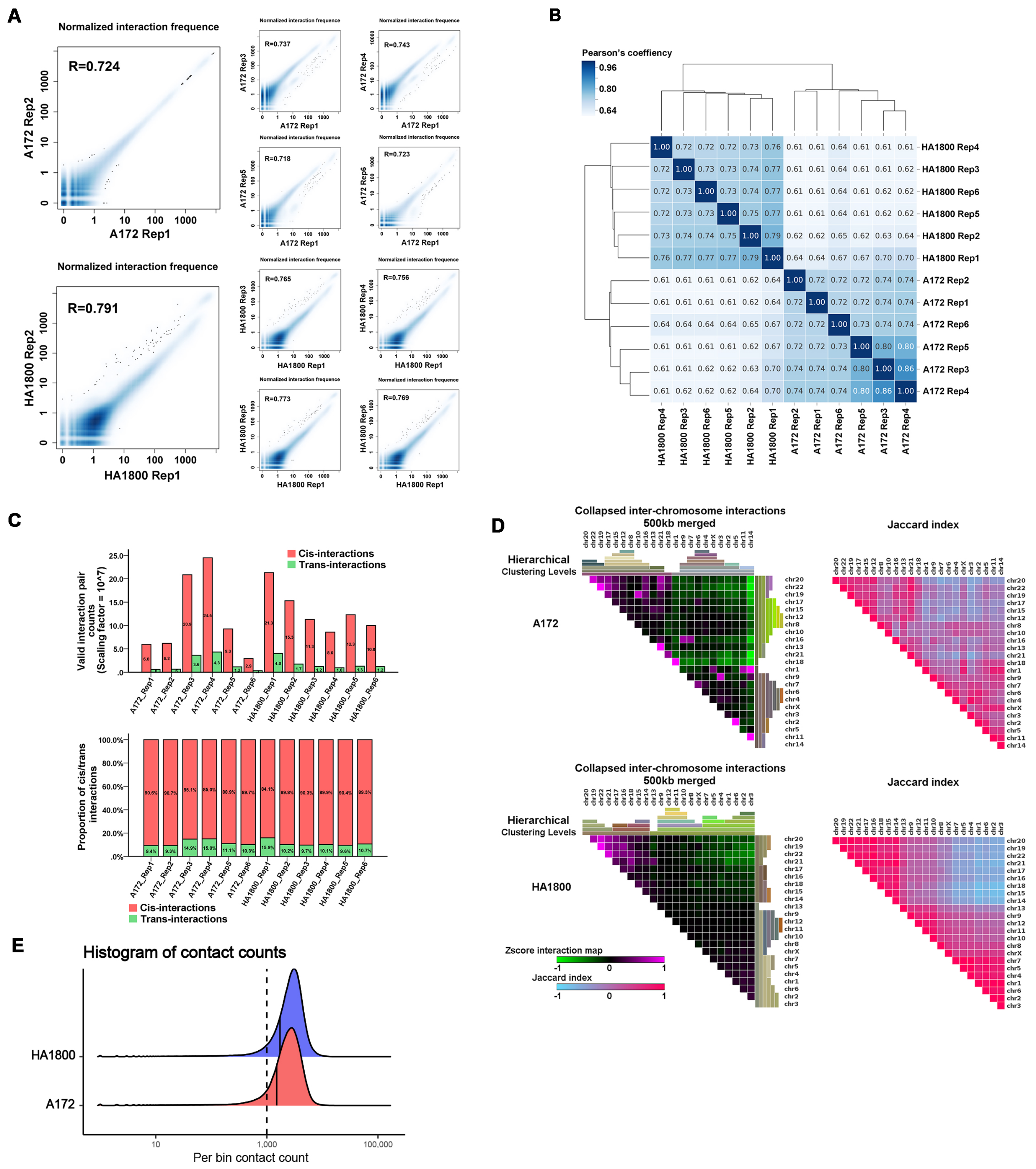
A
Normalized interaction frequence
R=0.724
A172 Rep2
A172 Rep1
R=0.737
A172 Rep3
R=0.743
A172 Rep4
R=0.718
A172 Rep5
R=0.723
A172 Rep6
R=0.791
HA1800 Rep2
HA1800 Rep1
R=0.765
HA1800 Rep3
R=0.756
HA1800 Rep4
R=0.773
HA1800 Rep5
R=0.769
HA1800 Rep6
B
Pearson's coeffiency
0.96
0.80
0.64
HA1800 Rep4
HA1800 Rep3
HA1800 Rep6
HA1800 Rep5
HA1800 Rep2
HA1800 Rep1
A172 Rep2
A172 Rep1
A172 Rep6
A172 Rep5
A172 Rep3
A172 Rep4
C
Cis-interactions
Trans-interactions
Valid interaction pair counts (Scaling factor = 10^7)
Proportion of cis/trans interactions
D
Collapsed inter-chromosome interactions 500kb merged
Hierarchical Clustering Levels
A172
HA1800
Jaccard index
Zscore interaction map
E
Histogram of contact counts
HA1800
A172
Per bin contact count

Sup. Figure 2

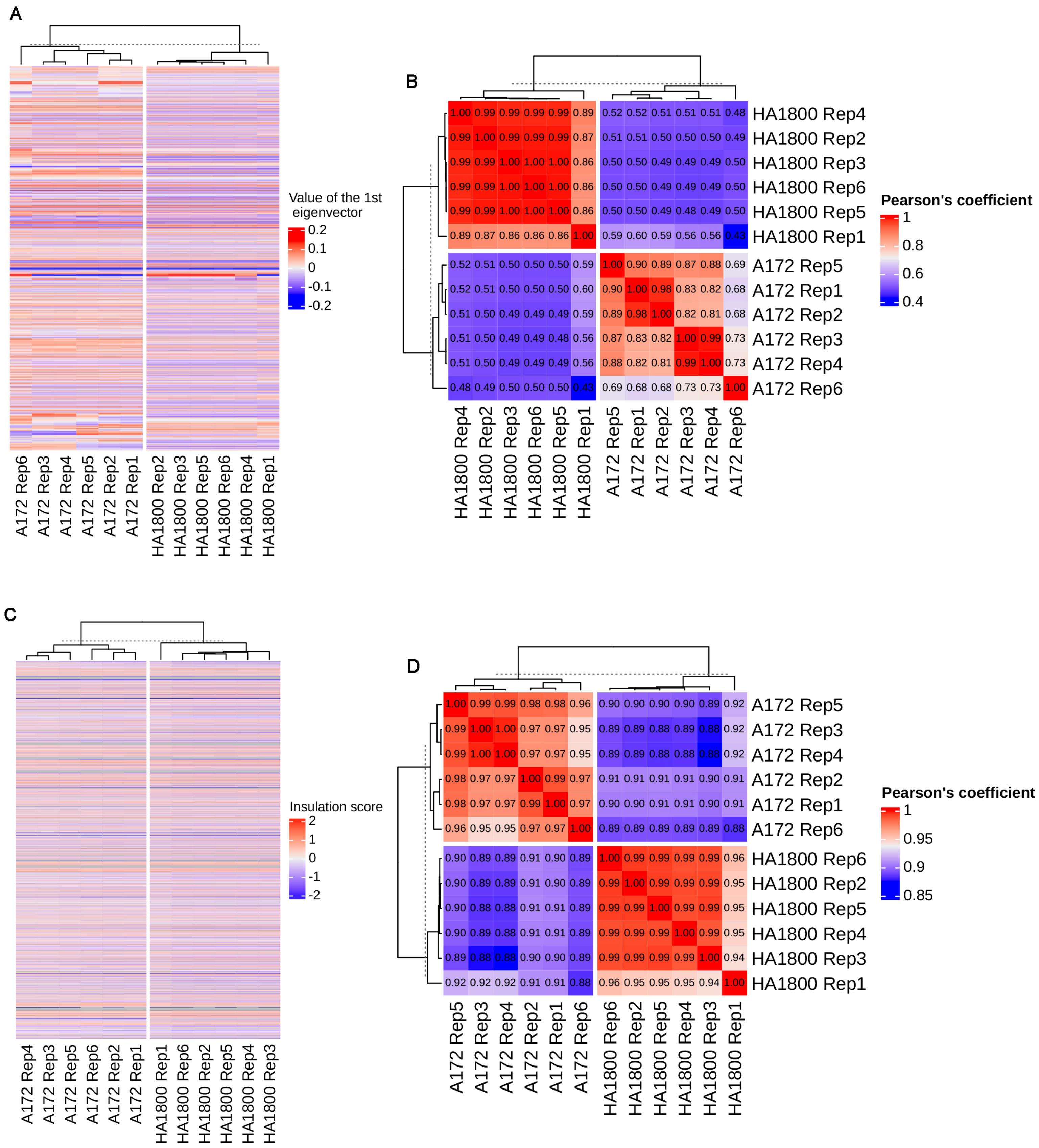
A
A172 Rep6
A172 Rep3
A172 Rep4
A172 Rep5
A172 Rep2
A172 Rep1
HA1800 Rep2
HA1800 Rep3
HA1800 Rep5
HA1800 Rep6
HA1800 Rep4
HA1800 Rep1
Value of the 1st eigenvector
0.2
0.1
0
-0.1
-0.2
B
HA1800 Rep4 1.00 0.99 0.99 0.99 0.99 0.89 0.52 0.52 0.51 0.51 0.51 0.48
HA1800 Rep2 0.99 1.00 0.99 0.99 0.99 0.87 0.51 0.51 0.50 0.50 0.50 0.49
HA1800 Rep3 0.99 0.99 1.00 1.00 1.00 0.86 0.50 0.50 0.49 0.49 0.49 0.50
HA1800 Rep6 0.99 0.99 1.00 1.00 1.00 0.86 0.50 0.50 0.49 0.49 0.49 0.50
HA1800 Rep5 0.99 0.99 1.00 1.00 1.00 0.86 0.50 0.50 0.49 0.48 0.49 0.50
HA1800 Rep1 0.89 0.87 0.86 0.86 0.86 1.00 0.59 0.60 0.59 0.56 0.56 0.43
A172 Rep5 0.52 0.51 0.50 0.50 0.50 0.59 1.00 0.90 0.89 0.87 0.88 0.69
A172 Rep1 0.52 0.51 0.50 0.50 0.50 0.60 0.90 1.00 0.98 0.83 0.82 0.68
A172 Rep2 0.51 0.50 0.49 0.49 0.49 0.59 0.89 0.98 1.00 0.82 0.81 0.68
A172 Rep3 0.51 0.50 0.49 0.49 0.48 0.56 0.87 0.83 0.82 1.00 0.99 0.73
A172 Rep4 0.51 0.50 0.49 0.49 0.49 0.56 0.88 0.82 0.81 0.99 1.00 0.73
A172 Rep6 0.48 0.49 0.50 0.50 0.50 0.43 0.69 0.68 0.68 0.73 0.73 1.00
HA1800 Rep4
HA1800 Rep2
HA1800 Rep3
HA1800 Rep6
HA1800 Rep5
HA1800 Rep1
A172 Rep5
A172 Rep1
A172 Rep2
A172 Rep3
A172 Rep4
A172 Rep6
Pearson's coefficient
1
0.8
0.6
0.4
C
A172 Rep4
A172 Rep3
A172 Rep5
A172 Rep6
A172 Rep2
A172 Rep1
HA1800 Rep1
HA1800 Rep6
HA1800 Rep2
HA1800 Rep5
HA1800 Rep4
HA1800 Rep3
Insulation score
2
1
0
-1
-2
D
A172 Rep5 1.00 0.99 0.99 0.98 0.98 0.96 0.90 0.90 0.90 0.90 0.89 0.92
A172 Rep3 0.99 1.00 1.00 0.97 0.97 0.95 0.89 0.89 0.88 0.89 0.88 0.92
A172 Rep4 0.99 1.00 1.00 0.97 0.97 0.95 0.89 0.89 0.88 0.88 0.88 0.92
A172 Rep2 0.98 0.97 0.97 1.00 0.99 0.97 0.91 0.91 0.91 0.91 0.90 0.91
A172 Rep1 0.98 0.97 0.97 0.99 1.00 0.97 0.90 0.90 0.91 0.91 0.90 0.91
A172 Rep6 0.96 0.95 0.95 0.97 0.97 1.00 0.89 0.89 0.89 0.89 0.89 0.88
HA1800 Rep6 0.90 0.89 0.89 0.91 0.90 0.89 1.00 0.99 0.99 0.99 0.99 0.96
HA1800 Rep2 0.90 0.89 0.89 0.91 0.90 0.89 0.99 1.00 0.99 0.99 0.99 0.95
HA1800 Rep5 0.90 0.88 0.88 0.91 0.91 0.89 0.99 0.99 1.00 0.99 0.99 0.95
HA1800 Rep4 0.90 0.89 0.88 0.91 0.91 0.89 0.99 0.99 0.99 1.00 0.99 0.95
HA1800 Rep3 0.89 0.88 0.88 0.90 0.90 0.89 0.99 0.99 0.99 0.99 1.00 0.94
HA1800 Rep1 0.92 0.92 0.92 0.91 0.91 0.88 0.96 0.95 0.95 0.95 0.94 1.00
A172 Rep5
A172 Rep3
A172 Rep4
A172 Rep2
A172 Rep1
A172 Rep6
HA1800 Rep6
HA1800 Rep2
HA1800 Rep5
HA1800 Rep4
HA1800 Rep3
HA1800 Rep1
Pearson's coefficient
1
0.95
0.9
0.85

Sup. Figure 3

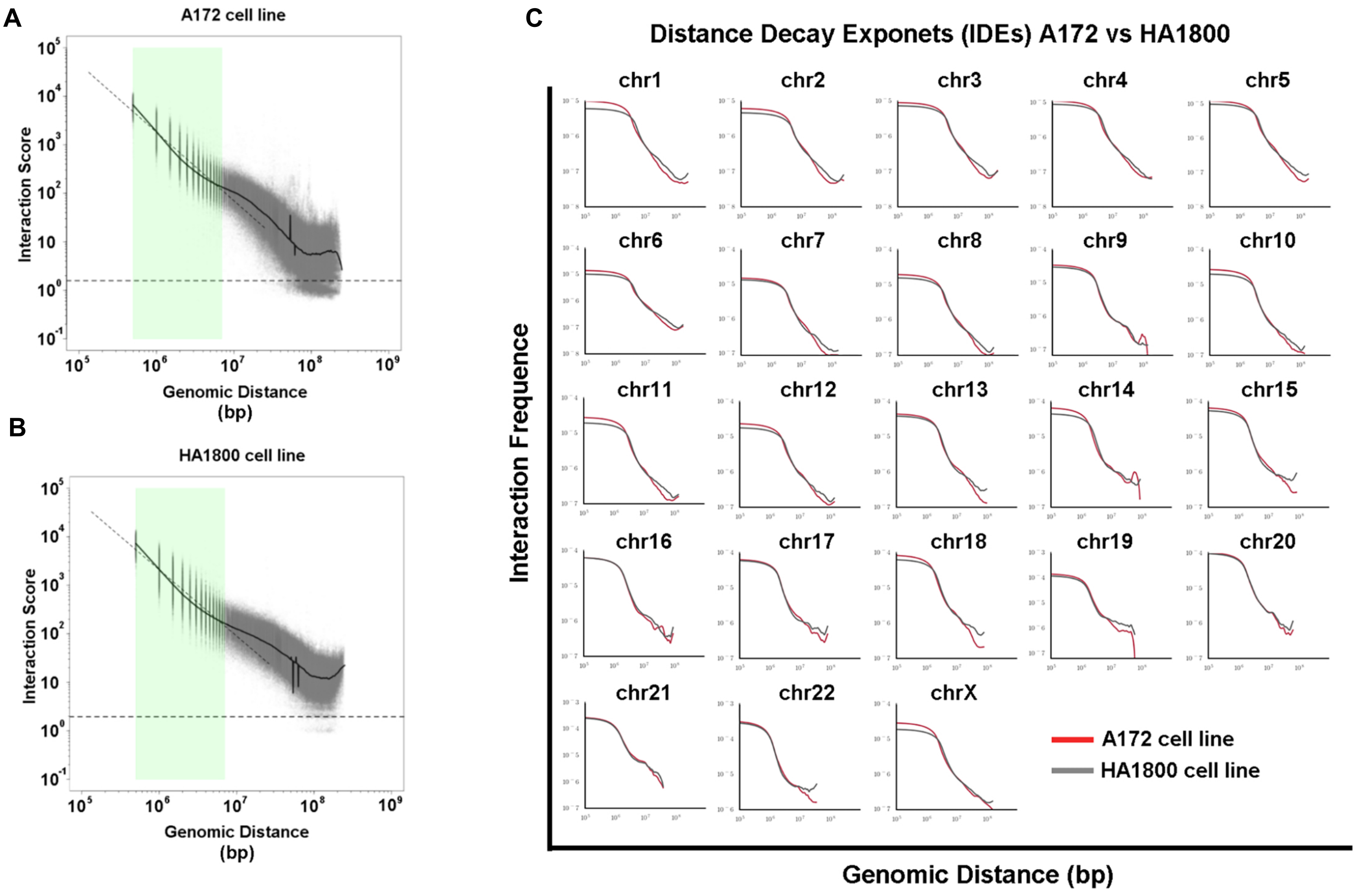
A
A172 cell line
Interaction Score
Genomic Distance
(bp)
B
HA1800 cell line
Interaction Score
Genomic Distance
(bp)
C
Distance Decay Exponets (IDEs) A172 vs HA1800
chr1
chr2
chr3
chr4
chr5
chr6
chr7
chr8
chr9
chr10
chr11
chr12
chr13
chr14
chr15
chr16
chr17
chr18
chr19
chr20
chr21
chr22
chrX
A172 cell line
HA1800 cell line
Interaction Frequence
Genomic Distance (bp)

Sup. Figure 4

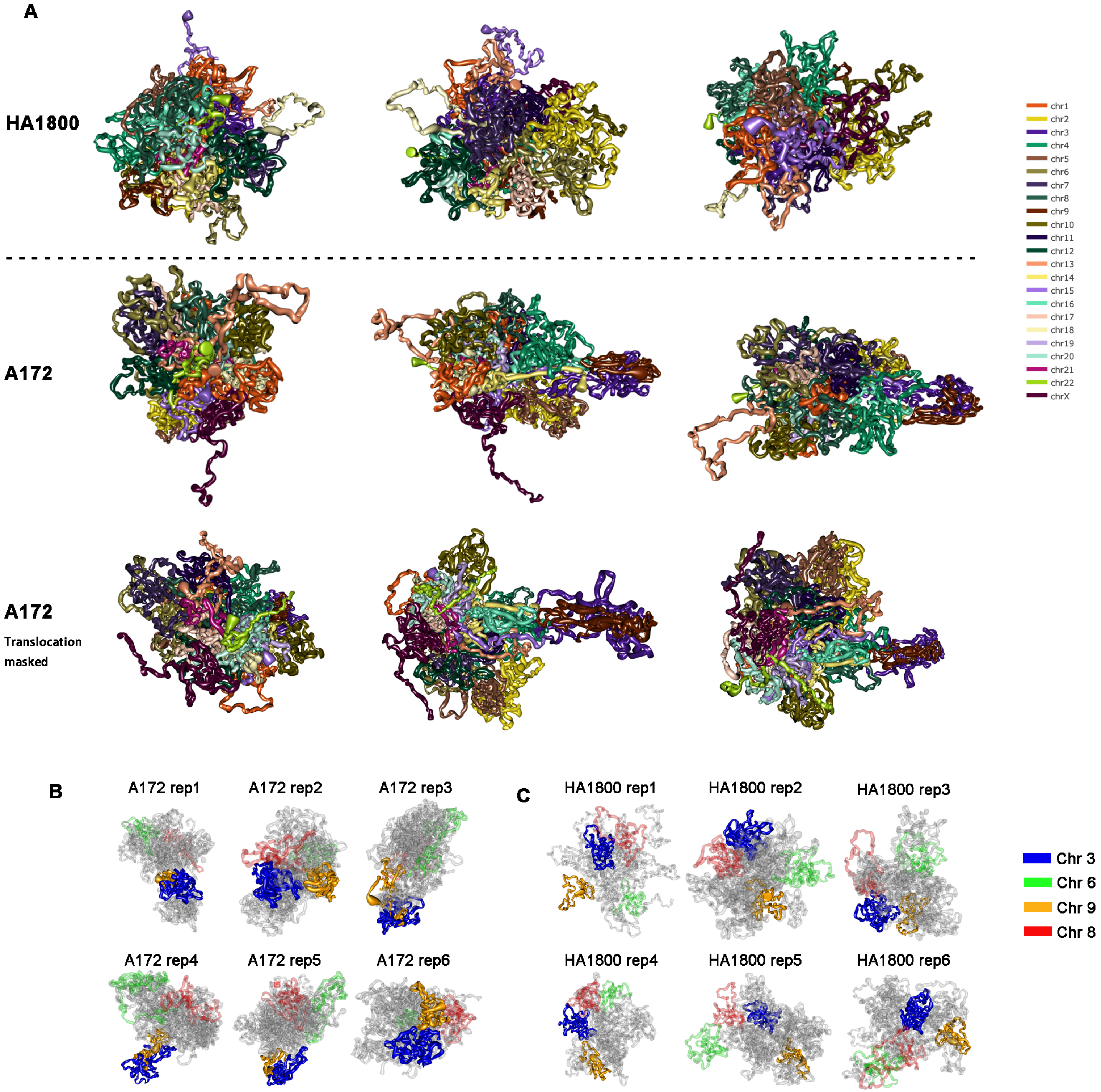
A
HA1800
A172
A172
Translocation masked
chr1
chr2
chr3
chr4
chr5
chr6
chr7
chr8
chr9
chr10
chr11
chr12
chr13
chr14
chr15
chr16
chr17
chr18
chr19
chr20
chr21
chr22
chrX
B
A172 rep1
A172 rep2
A172 rep3
A172 rep4
A172 rep5
A172 rep6
C
HA1800 rep1
HA1800 rep2
HA1800 rep3
HA1800 rep4
HA1800 rep5
HA1800 rep6
Chr 3
Chr 6
Chr 9
Chr 8

Sup. Figure 5

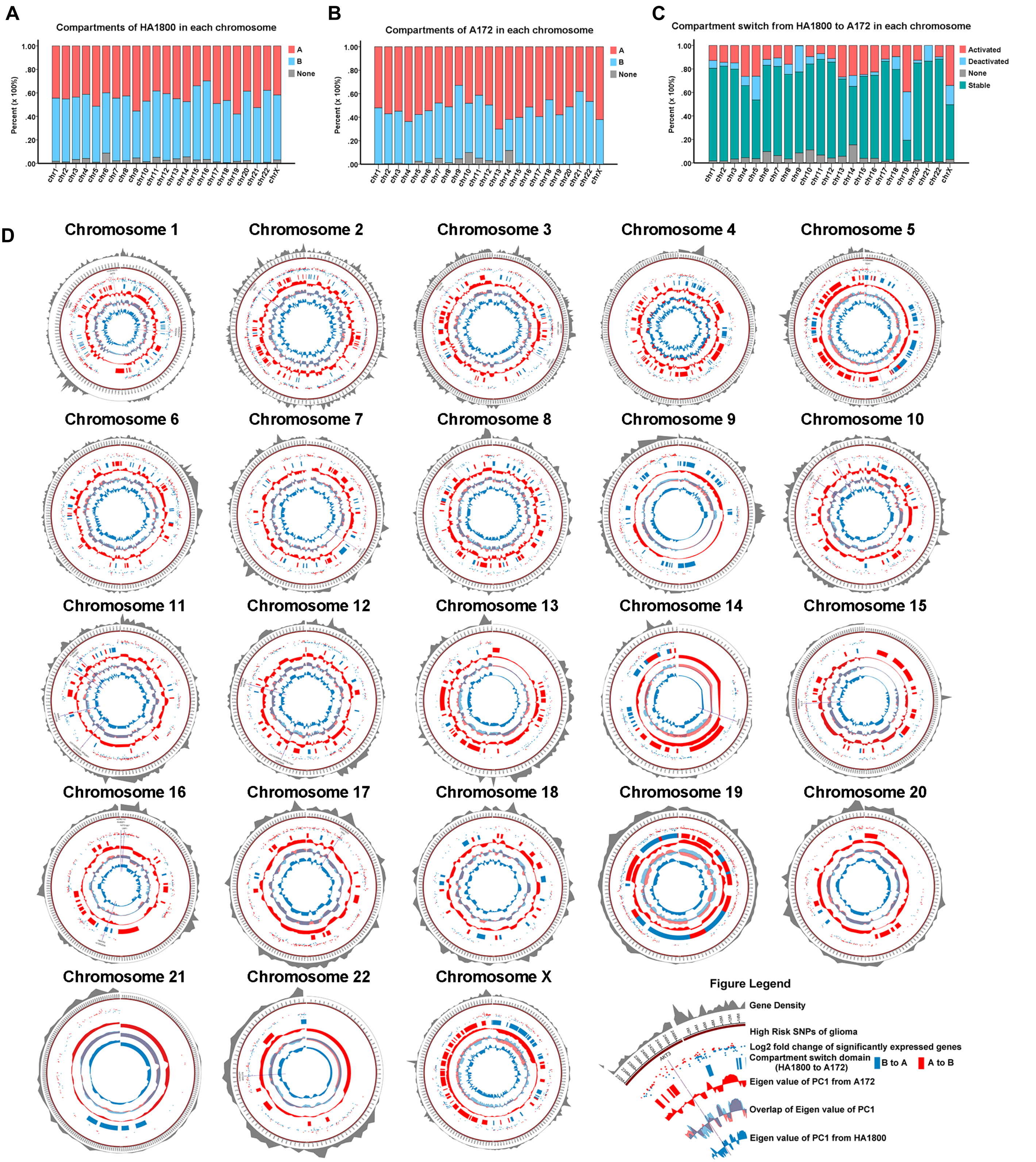
A
Compartments of HA1800 in each chromosome
B
Compartments of A172 in each chromosome
C
Compartment switch from HA1800 to A172 in each chromosome
Percent (x 100%)
A
B
None
Activated
Deactivated
Stable
D
Chromosome 1
Chromosome 2
Chromosome 3
Chromosome 4
Chromosome 5
Chromosome 6
Chromosome 7
Chromosome 8
Chromosome 9
Chromosome 10
Chromosome 11
Chromosome 12
Chromosome 13
Chromosome 14
Chromosome 15
Chromosome 16
Chromosome 17
Chromosome 18
Chromosome 19
Chromosome 20
Chromosome 21
Chromosome 22
Chromosome X
Figure Legend
Gene Density
High Risk SNPs of glioma
Log2 fold change of significantly expressed genes
Compartment switch domain (HA1800 to A172)
B to A
A to B
Eigen value of PC1 from A172
Overlap of Eigen value of PC1
Eigen value of PC1 from HA1800

Sup. Figure 6

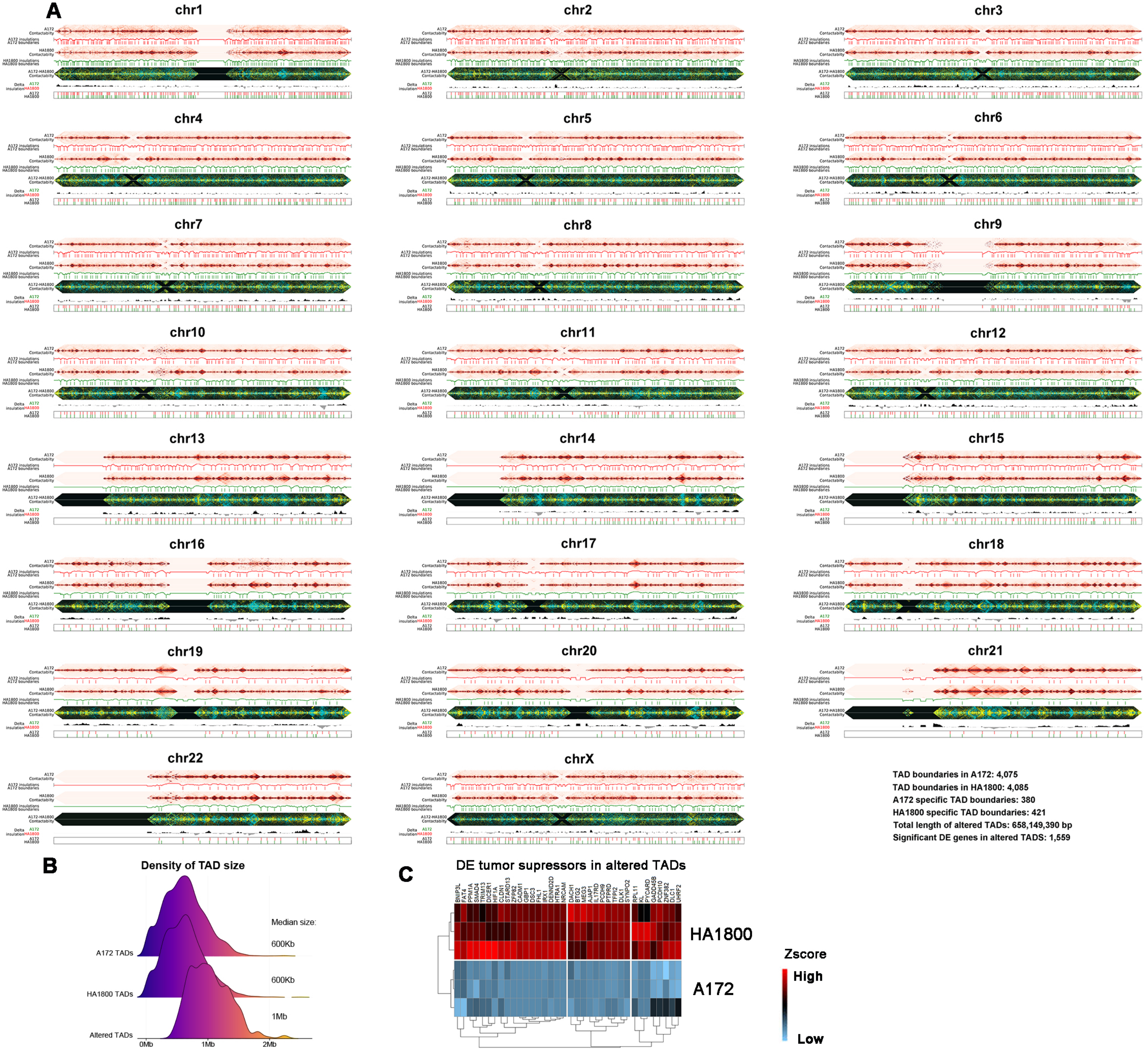
A
chr1
chr2
chr3
chr4
chr5
chr6
chr7
chr8
chr9
chr10
chr11
chr12
chr13
chr14
chr15
chr16
chr17
chr18
chr19
chr20
chr21
chr22
chrX
A172 Contactability
A172 insulations
A172 boundaries
HA1800 Contactability
HA1800 insulations
HA1800 boundaries
A172-HA1800 Contactability
Delta insulation
A172
HA1800
TAD boundaries in A172: 4,075
TAD boundaries in HA1800: 4,085
A172 specific TAD boundaries: 380
HA1800 specific TAD boundaries: 421
Total length of altered TADs: 658,149,390 bp
Significant DE genes in altered TADS: 1,559
B
Density of TAD size
Median size:
A172 TADs
600Kb
HA1800 TADs
600Kb
Altered TADs
1Mb
0Mb
1Mb
2Mb
C
DE tumor supressors in altered TADs
BNIP3L
FAT4
PPM1A
SMAD4
TRIM13
DICER1
HIF1A
CLDN1
STARD13
ZFP82
CADM1
GBP1
DSC3
FHL1
IRX1
DENND2D
HTRA1
NRCAM
DACH1
BTG2
MEG3
AJAP1
IL17RD
PCDH9
PTPRD
TFPI2
DLK1
SYNPO2
RPL11
KL
PYCARD
GADD45B
PCDH10
ZNF382
DLC1
UHRF2
HA1800
A172
Zscore
High
Low

Sup. Figure 7

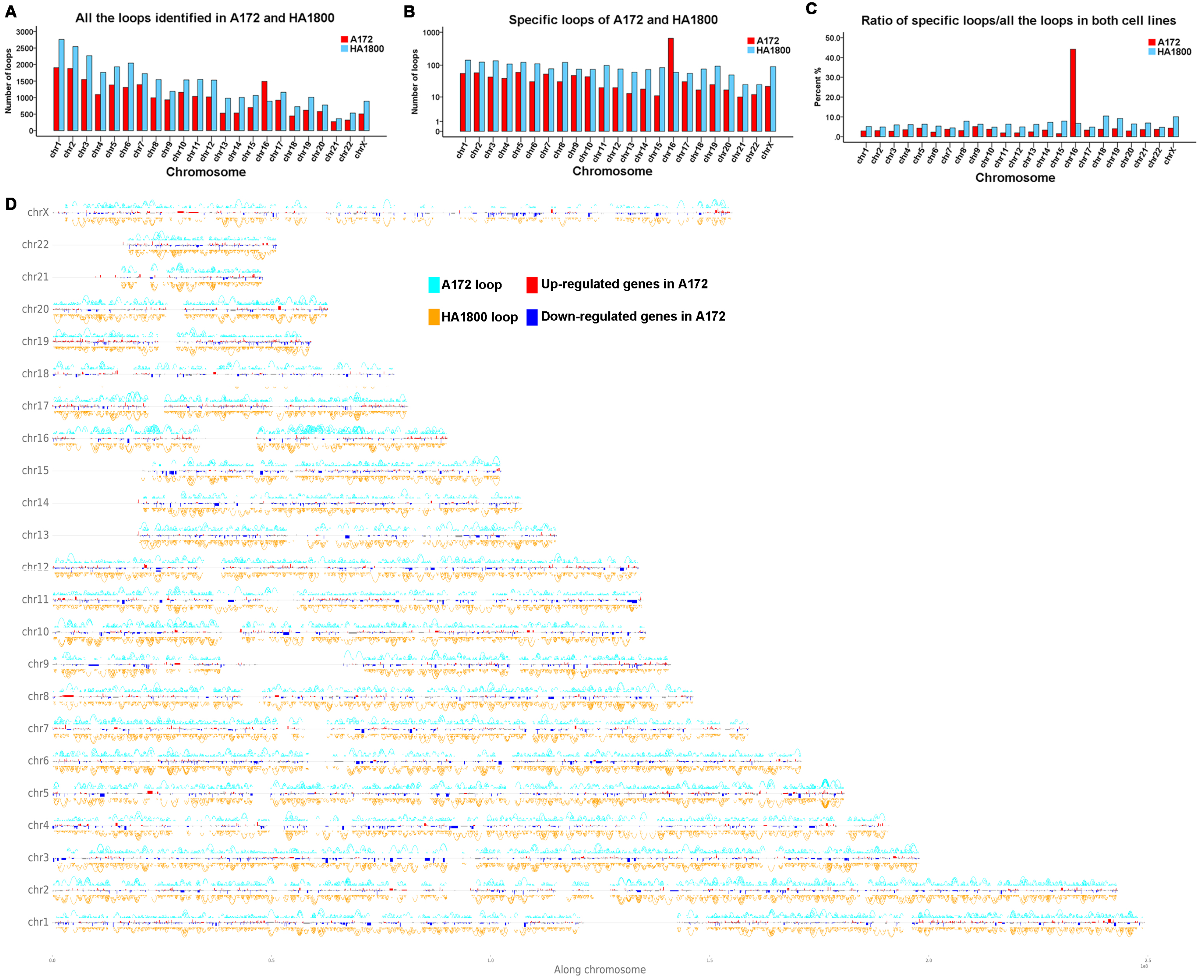
A
All the loops identified in A172 and HA1800
Number of loops
A172
HA1800
Chromosome
B
Specific loops of A172 and HA1800
Number of loops
Chromosome
C
Ratio of specific loops/all the loops in both cell lines
Percent %
Chromosome
D
A172 loop
HA1800 loop
Up-regulated genes in A172
Down-regulated genes in A172
Along chromosome
